# Trastuzumab deruxtecan in HER2-positive breast cancer with brain metastases: a single-arm, phase 2 trial

**DOI:** 10.1038/s41591-022-01935-8

**Published:** 2022-08-08

**Authors:** Rupert Bartsch, Anna Sophie Berghoff, Julia Furtner, Maximilian Marhold, Elisabeth Sophie Bergen, Sophie Roider-Schur, Angelika Martina Starzer, Heidrun Forstner, Beate Rottenmanner, Karin Dieckmann, Zsuzsanna Bago-Horvath, Helmuth Haslacher, Georg Widhalm, Aysegül Ilhan-Mutlu, Christoph Minichsdorfer, Thorsten Fuereder, Thomas Szekeres, Leopold Oehler, Birgit Gruenberger, Christian F. Singer, Ansgar Weltermann, Rainer Puhr, Matthias Preusser

**Affiliations:** 1 Department of Medicine I, Division of Oncology, Medical University of Vienna, Vienna, Austria; 2 Department of Radiology, Medical University of Vienna, Vienna, Austria; 3 Department Oncology, St. Joseph’s Hospital, Vienna, Austria; 4 Department of Radio-Oncology, Medical University of Vienna, Vienna, Austria; 5 Department of Pathology, Medical University of Vienna, Vienna, Austria; 6 Department of Laboratory Medicine, Medical University of Vienna, Vienna, Austria; 7 Department of Neurosurgery, Medical University of Vienna, Vienna, Austria; 8 Department of Oncology, LKH Wiener Neustadt, Wiener Neustadt, Austria; 9 Department of Gynaecology, Medical University of Vienna, Vienna, Austria; 10 Department of Medicine 1, Elisabethinen Hospital Linz, Ordensklinkum Linz, Linz, Austria

**Keywords:** Phase II trials, Breast cancer, CNS cancer

## Abstract

Trastuzumab deruxtecan is an antibody–drug conjugate with high extracranial activity in human epidermal growth factor receptor 2 (HER2)-positive metastatic breast cancer. We conducted the prospective, open-label, single-arm, phase 2 TUXEDO-1 trial. We enrolled patients aged ≥18 years with HER2-positive breast cancer and newly diagnosed untreated brain metastases or brain metastases progressing after previous local therapy, previous exposure to trastuzumab and pertuzumab and no indication for immediate local therapy. Patients received trastuzumab deruxtecan intravenously at the standard dose of 5.4 mg per kg bodyweight once every 3 weeks. The primary endpoint was intracranial response rate measured according to the response assessment in neuro-oncology brain metastases criteria. A Simon two-stage design was used to compare a null hypothesis of <26% response rate against an alternative of 61%. Fifteen patients were enrolled in the intention-to-treat population of patients who received at least one dose of study drug. Two patients (13.3%) had a complete intracranial response, nine (60%) had a partial intracranial response and three (20%) had stable disease as the best intracranial response, with a best overall intracranial response rate of 73.3% (95% confidential interval 48.1–89.1%), thus meeting the predefined primary outcome. No new safety signals were observed and global quality-of-life and cognitive functioning were maintained over the treatment duration. In the TUXEDO-1 trial (NCT04752059, EudraCT 2020-000981-41), trastuzumab deruxtecan showed a high intracranial response rate in patients with active brain metastases from HER2-positive breast cancer and should be considered as a treatment option in this setting.

## Abstract (web summary)

Findings from the TUXEDO-1 trial demonstrate efficacy of the antibody–drug conjugate trastuzumab deruxtecan for treatment of brain metastases in patients with HER2-positive breast cancer.

## Main

Brain metastases are a major burden in solid malignancies and of special concern in human epidermal growth factor receptor 2 (HER2)-positive breast cancer. Up to 15% of all patients with metastatic breast cancer will eventually develop brain metastases during their respective course of disease, making metastatic breast cancer the second most common cause of brain metastases among all solid tumors after lung cancer^1^, with the highest incidences reported in triple-negative and HER2-positive disease^2^. Overall, incidence of brain metastases has been rising over the last two decades, commonly attributed to improved overall survival due to the progress in systemic therapy options^3^ and a hypothetical shift to a more aggressive phenotype in patients recurring after primary adjuvant treatment^4^. Screening of asymptomatic patients and the ensuing diagnosis of asymptomatic brain metastases might further contribute to this observation.

Local therapy such as whole-brain radiotherapy (WBRT), stereotactic radiotherapy (SRT), stereotactic radiosurgery (SRS) and neurosurgery has been the mainstay of brain metastases treatment^5,6^, but patients’ prognosis remains generally poor with median overall survival times ranging from 2 months to 16 months, with outcomes differing by metastatic breast cancer subtype^1^. Overall survival in excess of 24 months was reported in patients with HER2-positive metastatic breast cancer brain metastases^7^. For these patients, systemic treatment has become an attractive alternative approach to WBRT when SRT or SRS is not possible or indicated, aiming at the prevention of WBRT-associated neurocognitive decline. Research in the field of systemic treatment has initially focused on small-molecule tyrosine-kinase inhibitors (TKIs) due to their low molecular mass. In contrast, larger molecules such as antibodies and antibody–drug conjugates (ADCs) were considered ineffective because of the blood–brain barrier (BBB). As the BBB is, however, disrupted at the site of metastases and replaced by a blood–tumor barrier with higher endothelial fenestration, larger molecules may also penetrate the brain parenchyma^8,9^. Indeed, ^64^Cu-tagged trastuzumab visualized brain metastases^10^ and several case series reported on the potential activity of the ADC T-DM1 (ado-trastuzumab emtansine) in breast cancer brain metastases^11,12^.

Trastuzumab deruxtecan (DS-8201a) is a new HER2-directed ADC consisting of a humanized HER2-directed monoclonal antibody (MAAL-9001) with the same amino acid sequence as trastuzumab, a cleavable molecular linker stable in plasma and deruxtecan, a topoisomerase-I inhibitor with high inhibitory potency and high membrane permeability^13,14^. The drug:antibody ratio in trastuzumab deruxtecan is 8:1 and therefore higher compared with earlier generation ADCs including T-DM1 (ref. ^14^). Trastuzumab deruxtecan was found to harbor clinically relevant activity in HER2-positive metastatic breast cancer progressing on previous T-DM1 (ref. ^15^) and prolonged progression-free survival (PFS) when directly compared with T-DM1 in the prospective randomized DESTINY-Breast03 study (hazard ratio (HR) 0.28, 95% confidence interval (CI) 0.22–0.37)^16^. Although outcomes were comparable in patients with stable brain metastases at baseline (HR = 0.25)^17^, data regarding the potential activity of trastuzumab deruxtecan in active brain metastases are limited. Therefore, the prospective, single-arm, single-center, phase 2 TUXEDO-1 trial was initiated. The study was specifically designed to evaluate efficacy and safety of trastuzumab deruxtecan in a population of HER2-positive breast cancer patients with active brain metastases (that is, newly diagnosed brain metastases or brain metastases progressing after previous local therapy) and more broadly as proof of principle for the intracranial activity of ADCs.

## Results

### Patient characteristics

Between 30 July 2020 and 23 July 2021, a total of 15 planned patients (14 women, 1 man) had received at least one cycle of trastuzumab deruxtecan; 60% had brain metastases progressing after previous local therapy and 60% had received previous T-DM1. The median time from the last local intervention to study inclusion in patients with previous local therapy was 13 months (range 5–65 months). The median age on inclusion was 69 years (30–76 years), Eastern Cooperative Oncology Group (ECOG) performance status was 0 in 60% of patients and 40% had neurological symptoms at baseline. Twelve patients had hormone-receptor-positive and HER2-positive disease and brain-only disease was present in two participants. All patient characteristics are summarized in Table 1. One patient initially assessed as having parenchymal brain metastases, and therefore included, was found to have dural metastasis on restaging and was therefore included in the primary endpoint analysis of response rate in the intention-to-treat (ITT) population and the safety population, but excluded from further efficacy analyses.

**Table 1:** Patient characteristics at baseline^a^

Characteristic	*N* = *15*
**Sex:** ***n*** **(%)**	
Female	14 (93.3)
Male	1 (6.7)
**Age: median (range)**	
Age at baseline (years)	69 (30–76)
**ECOG performance status:** ***n*** **(%)**	
ECOG 0	9 (60)
ECOG 1	6 (40)
**Presence of neurological symptoms at baseline:** ***n*** **(%)**	
Yes	6 (40)
No	9 (60)
**Disease subtype:** ***n*** **(%)**	
HER2-positive/luminal B	12(80)
HER2-positive/nonluminal	3 (20)
**Disease stage at primary diagnosis:** ***n*** **(%)**	
Stage IV	10 (66.7)
Stage I–III	5 (33.3)
**Brain metastasis-free survival (BMFS): median (range)**	
BMFS from diagnosis of metastatic disease (months)	17 (0-48)
**Brain-only disease:** ***n*** **(%)**	
Yes	2 (13.3)
No	13 (86.7)
**Visceral metastases:** ***n*** **(%**)	
Yes	12 (80)
No	3 (20)
**GPA index**^b^ **at baseline:** ***n*** **(%)**	
GPA 2.5	3 (20)
GPA 3.0	11 (73.3)
GPA 3.5	1 (6.7)
**Previous HER2-directed therapy:** ***n*** **(%)**	
Trastuzumab + pertuzumab	15 (100)
T-DM1	9 (60)
Lapatinib	4 (26.7)
Other	1 (6.7)
**Status of brain metastases;** ***n*** **(%)**	
Untreated	6 (40)
Primary brain metastases after previous local therapy	9 (60)
**Type of previous local therapy for brain metastases:** ***n*** **(%)**	
WBRT	3 (20)
WBRT + SRT/SRS and/or neurosurgery	3 (20)
SRT/SRS	3 (20)
**Time from last previous local intervention to inclusion: median (range)**	
Time from last local treatment (months)	13 (5–65)
**Previous lines of treatment for metastatic breast cancer: median (range)**	
Number of previous lines of treatment before trastuzumab deruxtecan	2 (1–5)

^a^*N*, Number of patients in the ITT population; *n*, number of patients.

^b^GPA, Graded Prognostic Assessment, breast cancer specific. Sparduto P. W. et al. Beyond an updated Graded Prognostic Assessment (Breast GPA): a prognostic index and trends in treatment and survival in breast cancer brain metastases from 1985 to today. *Int. J. Radiat. Oncol. Biol. Phys*. **107**, 334–343 (2020).

Four patients were pre-screened for study participation, but were not enrolled, because they did not fulfill the eligibility criteria (Methods). One had progressive brain metastases but had no measurable lesion by response assessment in neuro-oncology brain metastases (RANO-BM) criteria (that is, the largest lesion was <1 cm in diameter), two patients did not have progressive disease and one patient declined study participation and therefore received local therapy alone.

**Fig. 1:**
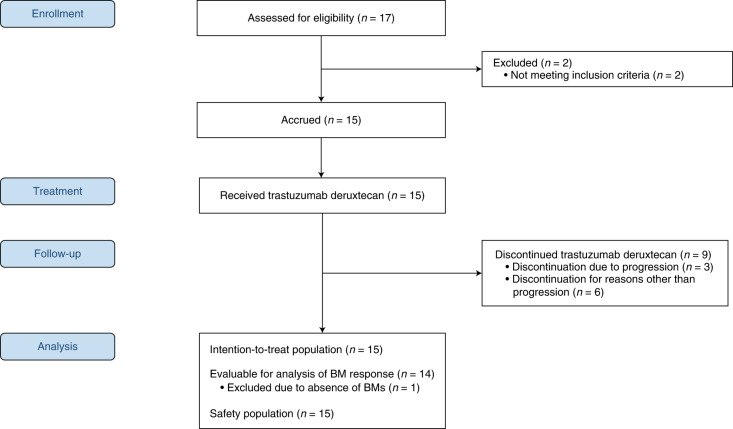
CONSORT flow diagram of the TUXEDO-1 trial. BM, brain metastases.

A consolidated standards of reporting trials (CONSORT) diagram is provided in Fig. 1.

### Efficacy

#### Primary outcome analysis

At the cut-off of 29 December 2021, 15 patients had received a total number of 170 cycles of trastuzumab deruxtecan. Median follow-up was 12 months (95% CI 8 months to not recorded). In the first stage of this trial (stage I enrollment between 30 July 2020, and 31 November 2020), responses by RANO-BM scores were observed in five of the six planned participants, and therefore the study progressed to the second stage with the planned accrual of an additional nine patients to a total number of fifteen patients (stage II enrollment between 1 December 2020 and 23 July 2021).

**Fig. 2:**
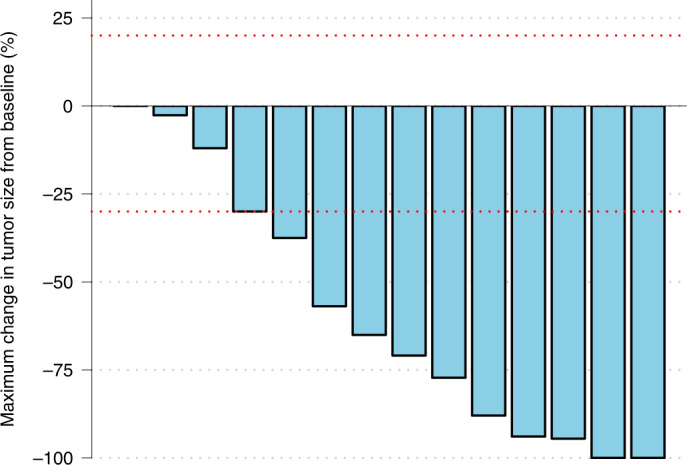
Waterfall plot of responses in patients evaluable for response by RANO-BM criteria in the TUXEDO-1 trial. Blue bars illustrate the radiographic change of maximum brain metastasis size after start of trastuzumab deruxtecan therapy compared to the baseline measurement. Red dotted lines denote thresholds for response and progression by RANO-BM criteria.

In the ITT population (*n* = 15 patients), intracranial response rate by RANO-BM was 73.3% (95% CI 48.1–89.1%) (11/15 patients; 2 patients in complete remission (13.3%); 9 patients in partial remission (60%)). In the per protocol population (PP; *n* = 14 patients), the response rate was 78.6% (95% CI 49.2–95.3%) (11/14). Two patients had stable disease for ≥6 months and one patient had stable disease at first restaging and progressed after four cycles of trastuzumab deruxtecan. Clinical benefit rate was 13/14 (92.9%; 95% CI 66.1–99.8%) in the PP population. The response outcomes of all 14 evaluable patients are shown in Fig. 2.

#### Secondary outcome analyses

In patients with extracranial metastases at baseline (*n* = 13), a partial response by RECIST 1.1 was observed in 5/13 (27.8%; 95% CI 13.9–68.4%) patients, with the remainder having stable disease. None of the patients progressing on trastuzumab deruxtecan had extracranial progression as the first site of progressive disease. In patients with measurable extracranial disease at baseline (*n* = 8), a partial remission was observed in 5/8 (62.5%; 95% CI 24.5–91.5%) patients, with the remainder having stable disease.

**Fig. 3:**
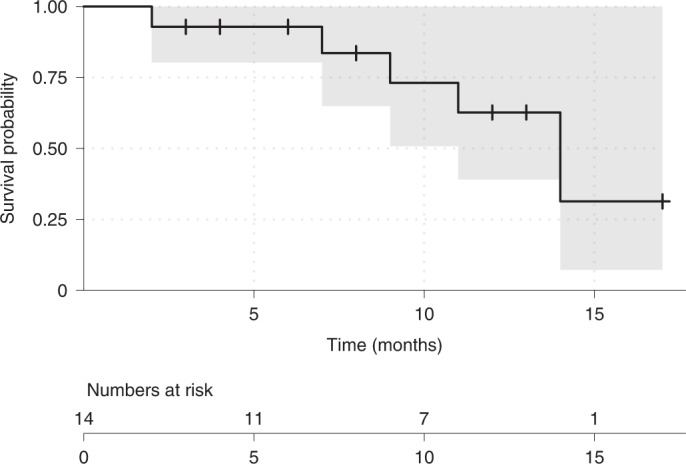
Kaplan–Meier plot showing progression-free survival times (months) in the TUXEDO-1 trial.

The median PFS was 14 months (95% CI 11.0 months to not recorded) (Fig. 3), irrespective of previous local therapy for brain metastases, previous T-DM1, hormone-receptor status, ECOG performance status and Graded Prognostic Assessment (GPA); the median overall survival was not reached, and three patients had died at the 12-month median follow-up. Reasons for treatment discontinuation were disease progression in three patients, treatment delay longer than allowed by protocol (two patients), interstitial lung disease (one patient), left-ventricular ejection fraction (LVEF) drop (one patient), serious adverse event (SAE; one patient) and patient’s wish (one patient). Six patients were still on treatment at the time of data cut-off. One patient died from urosepsis while on treatment and two patients had died from disease progression. As none of the patients with intracranial progression received WBRT as the next consecutive intervention, time to WBRT was not evaluable.

### Safety

All 15 patients experienced at least one AE (100%). Most AEs were mild and moderate; the main grade 1/2 hematological toxicities were anemia (46.6%) and neutropenia (46.6%). Grade 1/2 nonhematological AEs observed in more than two patients were fatigue (66.7%), nausea (46.7%), alopecia (40%), constipation (40%), hypokalemia (40%), diarrhea (33.4%), bone pain (26.6%), dyspnea (26.6%), fall (20%), urinary tract infection (20%) and vomiting (20%). Grade 3 AEs consisted of anemia, ejection fraction decrease, diarrhea, urinary tract infection and dyspnea in one patient each. Two cases of grade 3 fatigue were recorded, and one patient died from grade 5 sepsis. All AEs observed in the TUXEDO-1 trial are listed in Table 2.

**Table 2:** Adverse events

SOC and PT^a^	*N* = *15*^a^				
No. of patients with at least one AE^b^	*n* = 15 (100%)^a^				
	Grade 1	Grade 2	Grade 3	Grade 4	Grade 5
	*n* (%)^a^	*n* (%)^a^	*n* (%)^a^	*n* (%)^a^	*n* (%)^a^
**Blood and lymphatic system disorders**					
Anemia	5 (33.3)	2 (13.3)	1 (6.7)		
Neutropenia	2 (13.3)	5 (33.3)			
Thrombopenia	1 (6.7)				
**Cardiac disorders**					
Ejection fraction decreased			1 (6.7)		
**Ear and labyrinth disorders**					
Tinnitus	2 (13.3)				
Vertigo	1 (6.7)				
**Eye disorders**					
Extraocular muscle paresis		2 (13.3)			
**Gastrointestinal disorders**					
Abdominal pain	1 (6.7)				
Constipation	4 (26.7)	2 (13.3)			
Diarrhea	1 (6.7)	4 (26.7)	1 (6.7)		
Esophageal obstruction		1 (6.7)			
Gastritis		1 (6.7)			
Gastroesophageal reflux disease		1 (6.7)			
Hemorrhoidal hemorrhage	1 (6.7)				
Nausea		7 (46.7)			
Oral dysesthesia	1 (6.7)				
Vomiting		3 (20%)			
**General disorders and administration site conditions**					
Edema of limbs		1 (6.7)			
Fatigue	3 (20)	7 (46.7)	2 (13.3)		
Fever	1 (6.7)				
**Infections and infestations**					
Lung infection		1 (6.7)			
Laryngitis		1 (6.7)			
Mucosal infection	2 (13.3)				
Sepsis					1 (6.7)
Shingles		1 (6.7)			
Thrush	1 (6.7)	1 (6.7)			
Urinary tract infection		3 (20)	1 (6.7)		
**Injury, poisoning and procedural complications**					
Fall	2 (13.3)	1 (6.7)			
**Investigations**					
Blood bilirubin increased	2 (13.3)				
Weight gain		1 (6.7)			
**Metabolism and nutrition disorders**					
Anorexia	2 (13.3)				
Hypokalemia	6 (40)				
**Musculoskeletal and connective tissue disorders**					
Bone pain	2 (13.3)	2 (13.3)			
**Nervous system disorders**					
Dysgeusia	1 (6.7)				
Headache	1 (6.7)				
Peripheral sensory neuropathy	1 (6.7)	1 (6.7)			
Seizure		1 (6.7)			
**Psychiatric disorders**					
Depression		1 (6.7)			
Insomnia		1 (6.7)			
**Respiratory, thoracic and mediastinal disorders**					
Cough	1 (6.7)	1 (6.7)			
Dyspnea		3 (20)	1 (6.7)		
Epistaxis	1 (6.7)				
Pneumonitis		1 (6.7)			
**Skin and subcutaneous tissue disorders**					
Alopecia	6 (40)				
Palmar–plantar erythrodysesthesia syndrome	1 (6.7)				
Abscess		2 (13.3)			
Maculopapular rash	1 (6.7)				
**Vascular disorders**					
Thromboembolic event	1 (6.7)	1 (6.7)			

^a^SOC, system organ class; PT, preferred term; *N*, number of patients in the safety analysis set; *n*, number of patients.

^b^If a patient experienced >1 for a given AE, the patient was counted only once for the most severe grade.

A dose reduction by one step was recorded in five patients (33.3%) and two dose reductions were required in four patients (26.7%). Reasons for dose reduction were fatigue (four patients), diarrhea (three patients), neutropenia (one patient) and patient’s wish (one patient), respectively. Dose delays were necessary in three patients (neutropenia, left-ventricular systolic dysfunction and urinary tract infection in one patient each). A total of six SAEs was recorded in four patients. A full list of all SAEs is provided in Table 3. With regard to AEs of special interest, grade 2 interstitial lung disease and a symptomatic drop of LVEF were observed in one patient each.

**Table 3:** Serious adverse events^a^

SOC and PT^bb^	*N* = 15^b^
	*n* (%)^b^
Number of patients with at least one SAE	4 (26.7)
**Cardiac disorders**	
Ejection fraction decreased	1 (6.7)
**Infections and infestations**	
Lung infection	1 (6.7)
Urinary tract infection	1 (6.7)
Sepsis	1 (6.7)
**Musculoskeletal and connective tissue disorders**	
Pain	1 (6.7)
**Nervous system disorders**	
Seizure	1 (6.7)

^a^SAE: any AE resulting in death; immediately life threatening; requiring inpatient hospitalization or prolongation of hospitalization; resulting in persistent or substantial disability/incapacity; a congenital anomaly/birth defect in a child whose parent has been exposed to a medicinal product before conception or during pregnancy or is considered otherwise medically substantial, such as important medical events that may not immediately be life threatening or result in death or hospitalization, but jeopardize the subject or require intervention to prevent one of the outcomes listed in the definition above.

^b^SOC, system organ class; PT, preferred term; *N*, number of patients in the safety analysis set; *n*, number of patients.

### Quality of life

**Extended Data Fig. 1:**
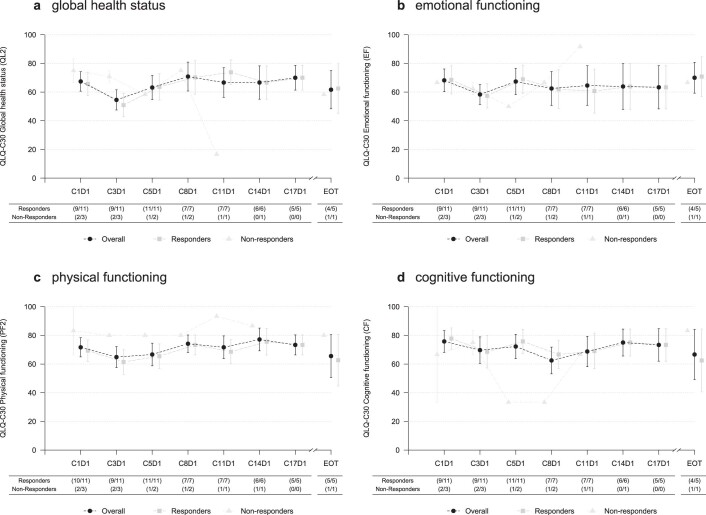
Health-related quality-of-life. Health-related quality-of-life. Data are presented as mean values +/- SEM. At each cycle data are based on (n/N) participants where n denotes the number of participants completing a section of the questionnaire at a specific cycle and N the total number of participants reaching that cycle.

Among the 14 patients with brain metastases who are eligible for health-related quality-of-life (QoL) assessment, 13 completed one or more assessments. A baseline assessment is available from 13 of 14 patients with parenchymal brain metastases and any follow-up assessment from all patients. As not all patients had filled in all forms completely, full sets with all QoL dimensions are available for nine patients only. The European Organisation for Research and Treatment of Cancer QoL core questionnaire, QLC-C30, global health status was maintained over the treatment period in the overall population; a drop in the nonresponders is probably caused by the small patient numbers in this group (Extended Data Fig. 1a). With regard to emotional and physical functioning, a numerical drop of QoL was observed from cycle 1, day 1 to cycle 3, day 1, but QoL improved to baseline levels thereafter (Extended Data Fig. 1b,c). With regard to cognitive functioning, maintained QoL levels were observed over the entire treatment period (Extended Data Fig. 1d).

### Post-hoc subgroup analysis

In patients with de novo brain metastases, the response rate was 100% compared with 66.7% in patients with brain metastases progressing after previous local therapy (PP population; Fisher’s exact test: *P* = 0.258). Exploratory analysis of intracranial response by RECIST 1.1 criteria confirmed the RANO-BM assessment in 14 of 15 patients. One patient fulfilled ‘stable disease’ by RANO-BM criteria, but ‘partial response’ by RECIST 1.1.

### Exploratory biomarker analysis

**Extended Data Fig. 2:**
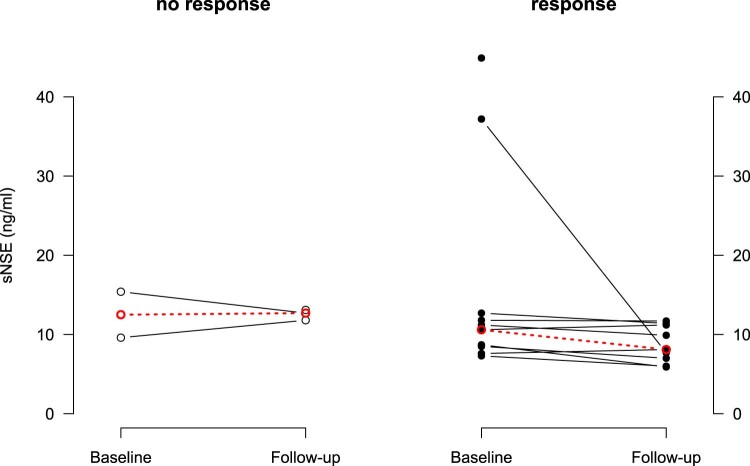
Neuron specific enolase levels. Neuron specific enolase levels (ng/ml) in patients with and without response to trastuzumab deruxtecan.

Serum neuron-specific enolase (sNSE) and serum S100 (sS100) levels were assessed in a total of 37 blood samples drawn at cycles 1 and 4 and end of treatment (EOT) in all patients of the ITT population (Methods). Matched samples were available from 11 patients. Median sNSE levels at baseline were 10.6 ng ml^−1^ (interquartile range (IQR) 8.6–12.2) in the responder group, compared with 12.5 ng ml^−1^ (IQR 12.2–12.9) in the nonresponder group, respectively (*P* = 0.621). Before the second radiological assessment (cycle 4), corresponding numbers were 8.1 ng ml^−1^ in patients responding to trastuzumab deruxtecan (IQR 7–11.2) and 12.7 ng ml^−1^ (IQR 12.2–12.9) in the nonresponder group, respectively (Mann–Whitney *U*-test; *P* = 0.009) (Extended Data Fig. 2). No differences in sS100 levels were observed between the responder and nonresponder groups at any time point (baseline *P* = 0.750 and follow-up *P* = 0.631, respectively).

## Discussion

The TUXEDO-1 trial is a prospective study reporting activity of an ADC in patients with active brain metastases. In a population of 15 patients with newly diagnosed or progressive brain metastases, trastuzumab deruxtecan yielded responses by RANO-BM criteria in 11 of 15 patients, for a response rate by central review of 73.3% in the ITT population. The median PFS was 14 months and median overall survival was not reached at a median follow-up of 12 months. These results indicate clinically relevant intracranial activity of trastuzumab deruxtecan and need to be discussed in the light of experimental and clinical data on systemic treatment options of brain metastases.

Single-agent therapy with the first-generation reversible HER2/EGFR (epidermal growth factor receptor) TKI lapatinib yielded a low intracranial response rate of 6% in patients with brain metastases progressing after previous local therapy in a phase 2 trial^18^. In an extension phase, treatment continuation on progression with the combination of lapatinib and capecitabine was allowed; in the present study, a promising response rate of 20% was observed with an additional 40% of patients having a minor reduction in the size of the brain metastases^18^. The same regimen was evaluated in the phase 2 LANDSCAPE trial conducted in a population of asymptomatic and oligosymptomatic patients with de novo brain metastases; Bachelot et al. reported a response rate of 66% and time to WBRT of 8.3 months^19^. In the TBCRC-022 trial, neratinib, a second-generation (irreversible) pan-HER2 TKI, in combination with capecitabine, yielded an intracranial response rate of 49% in patients with progressive brain metastases^20^. The HER2CLIMB trial randomized 612 patients progressing on previous therapy including trastuzumab, pertuzumab and T-DM1 to trastuzumab plus capecitabine with tucatinib, a third-generation HER2-specific TKI, or placebo^21^. Approximately half of the population had brain metastases at baseline (60% active brain metastases), making this by far the largest population of patients with active brain metastases accrued to a randomized trial to date. In the subset of patients with measurable brain metastases, the triple combination yielded a response rate of 47.3% compared with 20% in the control arm. Median PFS was improved from 4.1 months to 9.5 months (HR = 0.36, 95% CI 0.22–0.57) and overall survival from 11.6 months to 20.7 months (HR = 0.49, 95% CI 0.30–0.8). These data therefore firmly established the role of TKIs in the treatment of breast cancer brain metastases and the most current version of the respective European Society for Medical Oncology (ESMO) recommendations for the treatment of metastatic breast cancer and ASCO (American Society of Clinical Oncology)–SNO (Society for Neuro-Oncology)–ASTRO (American Society for Radiation Oncology) guidelines for the treatment of brain metastases has defined the combination of tucatinib, trastuzumab and capecitabine as the preferred systemic treatment option for active brain metastases from HER2-positive breast cancer^22–24^.

Despite the clear evidence from the HER2CLIMB study, the question of whether TKIs are generally superior to larger molecules for the systemic treatment of active brain metastases remains. Lapatinib was found not to pass through an intact BBB^25^ and the phase 3 CEREBEL trial could not establish a benefit of lapatinib over trastuzumab as prevention of brain metastases in pretreated metastatic breast cancer patients^26^. In addition, the BBB is impaired at the site of metastases, potentially allowing for the passage of molecules larger than TKIs into the brain parenchyma. As early as 1986, Rosner et al. reported on the activity of conventional chemotherapy as upfront therapy for breast cancer brain metastases^27^ and imaging studies could show that [^64^Cu]DOTA-trastuzumab visualizes brain metastases^10^. In prospective studies, however, response rates with antibody-based combination regimens, conducted in patients with progressive brain metastases, were disappointing^28,29^ and clinical data on the potential activity of ADCs in active brain metastases are limited. In a murine model of brain metastases, T-DM1 was shown to delay the growth of metastases compared with trastuzumab, resulting in a prolongation of overall survival^30^. In a small retrospective study of ten patients with de novo or progressive brain metastases, T-DM1 yielded an intracranial response by RANO-BM criteria of 30%^11^, in line with data from other case series^12^. The phase 3b KAMILLA trial allowed for the inclusion of patients with stable brain metastases at baseline. In the subset of patients with measurable brain metastases without previous local radiotherapy, the response rate with single-agent T-DM1 was 49.3%^31^. In the DESTINY-Breast03 study, trastuzumab deruxtecan yielded an objective intracranial response rate of 63.9% in patients with stable brain metastases at baseline^17^. The phase 2 DEBBRAH trial evaluated trastuzumab deruxtecan in different cohorts of patients with breast cancer and central nervous system disease. Preliminary results indicated activity in patients with stable brain metastases at baseline and responses were observed in five of nine patients with brain metastases progressing after previous local therapy^32^. A multi-institutional retrospective analysis of 16 breast cancer brain metastases patients treated with trastuzumab deruxtecan reported responses in 6 of 9 patients with progressive brain metastases as well^33^.

In summary, published data suggest that, despite their large molecular size, ADCs may achieve relevant clinical activity in brain metastases, potentially due to a partially disrupted BBB at the metastatic site. Trastuzumab deruxtecan was shown to harbor substantial extracranial activity in heavily pretreated HER2-positive metastatic breast cancer patients, and the DESTINY-Breast03 trial confirmed the superiority of trastuzumab deruxtecan over T-DM1, with the longest disease control ever observed in pretreated patients to date^16^. This observation is probably due to the specific pharmacological properties of trastuzumab deruxtecan that lead to a bystander effect with activity against neighboring HER2-negative cells. This latter assumption is of potential biological relevance because brain metastatic outgrowth requires close cooperation of cancer cells with astrocytes and the brain immune system^34^.

With a response rate of 73.3%, results from the TUXEDO-1 trial support the hypothesis of ADC activity in brain metastases and outcomes appear to be at least comparable to results of TKIs in a similar setting. The PFS of 14 months compares well with the 15-month PFS reported in the subset of patients with stable brain metastases at baseline in the DESTINY-Breast03 trial^17^ and is probably the longest PFS ever observed in a prospective trial evaluating systemic therapy in patients with active brain metastases to date. Due to the relatively short median follow-up of 12 months, PFS data should be interpreted with due caution.

We observed lower sNSE levels in patients responding to trastuzumab deruxtecan than in nonresponders at 4 weeks after treatment initiation, despite similar baseline levels. This finding may relate to reduced brain parenchyma destruction due to the inhibition of metastatic growth as a direct result of systemic therapy. Although this is an exploratory analysis, it could suggest a potential use of sNSE as a biomarker for monitoring of brain metastases in the clinical setting, which needs to be validated in further studies. At this stage, results need to be interpreted with due caution because the sample size is small and sNSE levels may be influenced by other factors as well.

With regard to toxicity, no new safety signals were observed and side effects were consistent with the toxicity profile expected from the pivotal trials. One patient was diagnosed with grade 2 interstitial lung disease and therefore had to permanently discontinue treatment, but recovered fully with systemic administration of corticosteroids. Global QoL and cognitive functioning were maintained over the duration of treatment. In conjunction with unparalleled extracranial disease control in the pivotal trials, our data suggest trastuzumab deruxtecan to be a safe and efficacious treatment option in patients with active brain metastases which may be preferred over TKI treatment, especially in the presence of additional extracranial disease burden.

Despite the strong biological rationale, the stringent response evaluation by RANO-BM criteria with central response assessment, the availability of biomarkers and extensive QoL evaluation, the present study is limited by the unrandomized phase 2 design and the small sample size and, thus, an inclusion bias cannot be fully excluded. TUXEDO-1 enrolled not only HER2-positive/estrogen receptor (ER)-negative tumors, but also HER2-positive/ER-positive cases, which show later recurrences and a less aggressive course of disease. However, response to trastuzumab deruxtecan therapies do not differ between these subtypes in the DESTINY-Breast01 trial and thus the overrepresentation of patients with luminal disease is not a likely reason for the high response rate observed in the study population^15^. Regardless, TUXEDO-1 is a prospective study indicating clinically relevant activity of the ADC trastuzumab deruxtecan in active brain metastases from HER2-positive breast cancer with comparable intra- and extracranial response rates in a pretreated population. In addition, the PFS results indicate prolonged disease control despite the presence of brain metastases. The results therefore suggest that trastuzumab deruxtecan could be safely used for the treatment of patients with active brain metastases from HER2-positive breast cancer, in case immediate local intervention is not indicated, and more generally support the notion that ADCs may be of interest in central nervous system malignancies and, thus, further clinical exploration in this context is warranted.

## Methods

TUXEDO-1 is an open-label, noncomparative, single-center, single-arm, phase 2 study evaluating the efficacy and safety of trastuzumab deruxtecan in HER2-positive breast cancer patients with newly diagnosed or progressing brain metastases who are deemed to be candidates for systemic therapy conducted at a tertiary care center. The present study was conducted in accordance with the Declarations of Helsinki and Good Clinical Practice and was approved by the local ethics committee of the Medical University of Vienna (EC no. 1359/2020). Written informed consent was obtained from each patient. None of the study participants received compensation for participation in the study. The trial is registered at ClinicalTrials.gov (NCT04752059) and the European Union Clinical Trials Register (EudraCT no. 2020-000981-41).

### Patients

To be eligible for inclusion in the TUXEDO-1 trial, each patient had to fulfill all of the following criteria: histologically confirmed breast cancer; radiologically documented metastatic disease; HER2-positive as defined by IHC3^+^ and/or HER2/neu^+^ gene amplification; newly diagnosed brain metastases or brain metastases progressing after previous local therapy; measurable disease as defined by RANO-BM criteria^35^; no indication for immediate local treatment; no indication of leptomeningeal disease; Karnofsky’s index of performance status (KPS) > 70%/ECOG < 2; indication for systemic anti-HER2 treatment; previous exposure to trastuzumab and pertuzumab; previous exposure to T-DM1 allowed; life expectancy of at least 3 months; age ≥18 years; patient able to tolerate therapy and have adequate cardiac function (defined by baseline LVEF ≥50%); adequate bone marrow, liver and kidney function; adequate treatment washout period before enrollment, defined as: major surgery ≥4 weeks, radiation therapy ≥4 weeks, chemotherapy, small-molecule targeted agents, anticancer hormonal therapy ≥3 weeks, antibody-based treatment ≥4 weeks; and patient capable of understanding the purpose of the study and has given written informed consent. Patients who fulfilled any of the following criteria were excluded: metastatic breast cancer other than HER2-positve disease; use of any investigational agent within 28 d before initiation of treatment; history of malignancy other than squamous cell carcinoma, basal cell carcinoma of the skin or carcinoma in situ of the cervix within the last 3 years, including contralateral breast cancer; major surgery, other than diagnostic surgery, within the last 4 weeks; indication for immediate local therapy by local standard; leptomeningeal involvement; other anticancer therapy, including cytotoxic, targeted agents, immunotherapy, antibody, retinoid or anticancer hormonal treatment; concomitant radiotherapy; previous radiotherapy to the thorax other than breast irradiation or irradiation of bone metastases; a history of uncontrolled seizures, central nervous system disorders or psychiatric disability judged by the investigator to be clinically significant and adversely affecting compliance to study drugs; clinically significant cardiac disease including unstable angina, acute myocardial infarction within 6 months before randomization, congestive heart failure (New York Heart Association III–IV), LVEF <50%, arrhythmia unless controlled by therapy, with the exception of extrasystole or minor conduction abnormalities, and long QT syndrome (QTc interval >450 ms); subjects who have current active hepatic or biliary disease (with the exception of patients with Gilbert’s syndrome, asymptomatic gallstones, liver metastases or stable chronic liver disease according to investigator assessment), including acute and chronic infections with hepatitis B and C; inadequate hematological status at baseline before study entry: dependency on red blood cell and/or platelet transfusions, absolute neutrophil count (segmented + bands) <1.0 × 10^9^ l^−1^; platelets <100 × 10^9^ l^−1^; inadequate kidney function: serum creatinine >1.5× upper normal limit; hepatic dysfunction: total bilirubin >1.5× upper normal limit (>3 in patients with liver metastases or known history of Gilbert’s disease); alanine transaminase, aspartate aminotransferase >3× upper normal limit (>5 in patients with liver metastases); serum albumin <2.5 g dl^−1^; international normalized ratio ≥1.5; clinically severe pulmonary compromise resulting from intercurrent pulmonary illnesses, including, but not limited to, any underlying pulmonary disorder (that is, pulmonary emboli within 3 months of the study enrollment, severe asthma, severe chronic obstructive pulmonary disease, restrictive lung disease, pleural effusion, and so on), and any autoimmune, connective tissue or inflammatory disorders with pulmonary involvement (that is, rheumatoid arthritis, Sjögren’s syndrome, sarcoidosis and so on) or previous pneumonectomy (subjects with bronchopulmonary disorders who require intermittent use of bronchodilators (such as albuterol) not excluded from the present study); patients with active opportunistic infections; known human immunodeficiency virus infection; concomitant treatment with chloroquine or hydroxychloroquine; and pregnant or lactating women. Women with childbearing potential must have a negative pregnancy test at screening; also excluded are women with childbearing potential, including women whose last menstrual period was <1 year before screening, unable or unwilling to use adequate contraception from study start to 1 year after the last dose of protocol therapy. Acceptable contraception methods included the application of an intrauterine device, barrier method or total abstinence. Also included are patients with known hypersensitivity to trastuzumab, patients unable to provide written informed consent, patients with known substance abuse or any other medical conditions such as clinically significant cardiac or psychological conditions, which may, in the opinion of the investigator, interfere with the subject’s participation in the clinical study or evaluation of the clinical study results, and patients requiring concomitant use of chronic systemic (intravenous or oral) corticosteroids at doses >4 mg of dexamethasone per day or other immunosuppressive medications except for managing AEs (inhaled steroids or intra-articular steroid injections are permitted in the present study).

### Study procedures

In this trial, trastuzumab deruxtecan was administered at the standard dose of 5.4 mg per kg bodyweight on day 1 of each cycle once every 3 weeks until progression, unacceptable toxicity or withdrawal for any other reason (Fig. 1). Before the first administration of trastuzumab deruxtecan, cranial magnetic imaging resonance, a bone scan and a computed tomography scan of the chest and abdomen were conducted with further workup if indicated. Staging investigations were to be repeated before the third and fifth treatment cycles and every 9 weeks thereafter, or whenever symptoms of disease progression occurred. The trial protocol provides a detailed overview of study procedures at baseline and during the study.

If patients progressed on study therapy, they entered survival follow-up. If treatment was discontinued for any reason other than progression and no alternative anticancer therapy was initiated and consent not withdrawn, patients were eligible for analysis of PFS without censoring at the time of study drug discontinuation. If alternative anticancer therapy was initiated at the time of trastuzumab deruxtecan discontinuation, patients entered survival follow-up. If patients were withdrawn within the first 9 weeks of the study (that is, before the first response evaluation) for reasons other than progression or death, they were replaced.

### Endpoints

The primary endpoint of the TUXEDO-1 study was the rate of best responses of brain metastases at any radiological assessment after the administration of at least one cycle of trastuzumab deruxtecan. Objective response was defined as complete remission, partial remission, stable disease and progressive disease according to the RANO-BM criteria, as determined by the central assessment of a single board-certified neuroradiologist^35^. Secondary endpoints consisted of: Clinical Benefit Rate in the central nervous system (CBR CNS as defined by RANO-BM; complete remission/partial remission/stable disease ≥6 months), extracranial response rate defined as complete remission, partial remission, stable disease, progressive disease according to RECIST 1.1 criteria^36^, PFS defined as the interval from study inclusion until progression or death, time to WBRT defined as the interval from study inclusion until WBRT, overall survival defined as the interval from study inclusion until death, safety and QoL as assessed with the EORTC QLQ-c30 questionnaire, the brain-specific tool (BN20), and the breast-specific tool (BR45). For ancillary biomarker studies, blood samples (3 ml of EDTA, 3 ml of serum) were drawn before first administration of trastuzumab deruxtecan at cycles 1 and 4 and at EOT. The biomarker substudy of TUXEDO-1 aimed to investigate changes in the extent of metastases-induced brain damage in patients with and without response to therapy by measuring the levels of sNSE and sS100, two proteins constitutively expressed in the human brain and established markers of brain damage in neuro-oncology and ischemic brain damage^37–39^. Both markers were quantified by means of electrochemiluminescence assays according to standard operating procedures on cobas e801 analyzers (Roche Diagnostics) by an International Organization for Standardization 15189:2012-accredited medical laboratory (Department of Laboratory Medicine, Medical University of Vienna).

### Statistical analysis

TUXEDO-1 was designed as a phase 2 study evaluating the ability of trastuzumab deruxtecan to induce objective responses in patients with HER2-positive metastatic breast cancer and with newly diagnosed or progressive brain metastases based on a Simon’s two-stage design^40^. A response rate of ≤25% was considered to be of no clinical interest whereas a response rate of ≥61% was considered to be clinically relevant. The null hypothesis that the true response rate was 25% was tested against a one-sided alternative. Based on these assumptions, six patients were to be accrued in the first stage. If at least three responses were observed in the first stage, nine additional patients were to be accrued for a total number of fifteen patients. The null hypothesis could be rejected if seven or more responses were observed in these fifteen patients. This design yields a type I error rate of 5% and a power of 80% to reject the null hypothesis when the true response rate is 61%.

The response rate was analyzed on the ITT principle, wherein all patients who had received at least one dose of the study drug were included in the analysis. Responses were summarized using frequency counts and percentages with 95% CIs. Fisher’s exact test was used for the comparison of differences in response rates between patients with newly diagnosed, untreated brain metastases and brain metastases progressing after previous local therapy. PFS and overall survival were estimated using the Kaplan–Meier product limit method.

Safety and tolerability of treatment in terms of hematological and nonhematological side effects were assessed by the investigators at each visit and graded according to the National Cancer Institute Common Terminology Criteria for Adverse Events (CTCAE) v.5.0. SAEs were defined according to the International Conference on Harmonization Good Clinical Practice guidelines. AEs were summarized using frequency counts and percentages. All patients who were eligible for the present study and received at least one dose of study drug were included in the safety analysis.

QoL was assessed at day 1 of cycles 1, 3 and 5 and every 9 weeks thereafter. A final QoL assessment was conducted at the first survival follow-up at 3 months after EOT. Changes from baseline were analyzed using a linear mixed-effect model and separately displayed for the overall patient population and for the respective responder and nonresponder groups. Data were expressed as the mean ± s.e.m.

For the biomarker substudy, sNSE and sS100 levels were compared between responders and nonresponders using Mann–Whitney *U*-tests. Two-sided P values <5% were considered statistically significant. No correction for multiple testing was performed due to the hypothesis-generating design of this biomarker substudy^41^.

Statistical analysis was conducted using R 4.1.3. and IBM SPSS Statistic v.28.

### Reporting summary

Further information on research design is available in the Nature Research Reporting Summary linked to this article.

## Online content

Any methods, additional references, Nature Research reporting summaries, extended data, supplementary information, acknowledgements, peer review information; details of author contributions and competing interests; and statements of data and code availability are available at 10.1038/s41591-022-01935-8.

## Supplementary information

Supplementary Information

Reporting Summary

## Data Availability

Pseudonymized participant data including baseline characteristics and results of primary, secondary and exploratory endpoint analyses reported in this article can be shared in compliance with current data protection regulations by the European Union. Data sharing requires a current and positive vote by the requestors competent ethics committee. All proposals should be directed to the corresponding author and data requestors will need to sign a data access agreement with the Medical University of Vienna.

## Extended data

are available for this paper at 10.1038/s41591-022-01935-8.
