# Supplementary material for: Trastuzumab deruxtecan in HER2-positive breast cancer with brain metastases: a single-arm, phase 2 trial
Source: Nat Med. 2022 Aug 8;28(9):1840–7. doi: 10.1038/s41591-022-01935-8 (PMC9499862; doi:10.1038/s41591-022-01935-8)
Supplement: Supplementary file 1 — Supplementary Information [file 41591_2022_1935_MOESM1_ESM.pdf]

nature portfolio

https://doi.org/10.1038/s41591-022-01935-8

Supplementary information

Trastuzumab deruxtecan in HER2-positive breast cancer with brain metastases: a single-arm, phase 2 trial

In the format provided by the
authors and unedited

Clinical Study Protocol

Phase II Study of Trastuzumab-Deruxtecan (T-DX; DS-8201a) in HER2-positive Breast Cancer Patients with newly diagnosed or progressing Brain Metastases

---

TUXEDO-1

Version 3.0/13.08.2020

EudraCT 2020-000981-41

Confidentiality Statement

The information contained in this document is the property of the sponsor of this trial and therefore is provided to you in confidence for review by you, your staff, an applicable Ethics Committee/Institutional Review Board and regulatory authorities. It is understood that the information will not be disclosed to others without prior written approval from the sponsor, except to the extent necessary to obtain informed consent from those persons to whom the medication may be administered.

**Test drug (IMP)**	**Trastuzumab-Deruxtecan (T-DXd; DS-8201a)**
**Protocol authors**	**Assoc.-Prof. PD Dr. Rupert Bartsch, Ass.-Prof. PD Dr. Anna Sophie Berghoff, Dr. Elisabeth Sophie Bergen, Mag. Ortrun Neuper, o.Univ. Prof. Matthias Preusser**
**Principal investigator**	**Assoc.-Prof. PD Dr. Rupert Bartsch,**<br>**Deptartment of Medicine I, Division of Oncology, Medical University of Vienna, Vienna General Hospital**<br>**Waehringer-Gürtel 18-20**<br>**1090 Vienna**<br>**email: rupert.bartsch@meduniwien.ac.at**
**Document type**	**Clinical Study Protocol**
**Study phase**	**Phase II**
**Version/Date**	**3.0/13.08.2020**
**Number of Pages**	**73**

SPONSOR, INVESTIGATOR AND SIGNATURES

TUXEDO-1, Version 3.0/13.08.2020

Sponsor/OEL

_________________ ___________

Signature **Date**

o. Univ. Prof. Dr. Herbert Watzke

Department of Medicine I, Medical University of Vienna, Austria,

Investigator

I have thoroughly read and reviewed the study protocol "Phase II Study of Trastuzumab-Dexruxtecan (T-DXd; DS-8201a) in HER2-positive Breast Cancer Patients with newly diagnosed or progressing Brain Metastases" Version 3.0_13.08.2020. Having read and understood the requirements and conditions of the study protocol, I agree to perform the clinical study according to the international good clinical practice principles and regulatory authority requirements.

I understand that changes to the protocol must be made in form of an official amendment.

I agree to report all serious adverse events, whether considered treatment-related or not within one working day.

_________________ ___________

Signature Date

Assoc.-Prof. PD Dr. Rupert Bartsch, Department of Medicine I,

Division of Oncology, Medical University of Vienna, Austria

PROTOCOL SYNOPSIS

TITLE	Phase II Study of Trastuzumab-Deruxtecan (T-DXd; DS-8201a) in HER2-positive Breast Cancer Patients with newly diagnosed or progressing Brain Metastases
ACRONYM	TUXEDO-1
INDICATION	Patients with histologically proven HER2-positive metastatic breast cancer and newly diagnosed or progressing brain metastases eligible for systemic treatment
SCIENTIFIC RATIONAL	Brain metastases (BM) are a common and devastating complication of HER2-positive breast cancer. For decades, local therapy such as whole brain radiotherapy (WBRT), radiosurgery and/or neurosurgical resection has been the mainstay of treatment. While WBRT usually yield response rates of around 50% and may alleviate neurological symptom, severe neurological late toxicity must be expected. This problem became evident with BM patients surviving for more than two years since the introduction of highly active systemic treatment.<br><br>In order to delay the need for WBRT, activity of upfront systemic treatment was evaluated within the LANDSCAPE trial of lapatinib plus capecitabine defining a potential novel treatment standard in patients with multiple oligo- or asymptomatic BM. More recently, promising data were published with the second- and third-generation tyrosine-kinase inhibitors neratinib and tucatinib and the antibody-drug conjugate T-DM1. As T-DM1 is increasingly used in the early-stage disease setting, further options for the treatment of metastatic disease are urgently required.<br><br>Trastuzumab-deruxtecan (DS-8201a) is a novel HER2-directed antibody-drug conjugate with high clinical activity in HER2-positive MBC and other HER2-overexpressing solid cancers. In addition, responses were also noted in patients with HER2-low expressing tumours. Of note, treatment was generally well tolerated with rare cases of pneumonitis observed. Thus, the promising clinical activity in conjunction with the safety profile renders this drug attractive for the treatment of patients with BM.<br><br>In this phase II study, activity and safety of trastuzumab-deruxtecan in patients with BM from HER2-positive breast cancer will be evaluated.

DESIGN / PHASE	Prospective, single centre, open label, non-comparative, phase II
STUDY DURATION	3 years
CENTER<br>/ COUNTRY	Medical University of Vienna, Department of Medicine I, Division of Oncology, Vienna, Austria
PATIENTS / GROUPS	15 evaluable patients overall; in stage 1, 6 patients will be initially accrued, with accrual of additional 9 patients up to 15 maximum patient population in stage 2 according to the number of responses seen in stage 1

INCLUSION CRITERIA	• Histologically confirmed breast cancer<br>• Radiologically documented metastatic disease<br>• HER2-positive as defined by IHC 3+ and/or *HER2/neu* gene amplification<br>• Newly diagnosed brain metastases or brain metastases progressing after prior local therapy<br>• Measurable disease (RANO-BM criteria)<br>• No indication for immediate local treatment<br>• No indication of leptomeningeal disease<br>• KPS >70%, ECOG <2<br>• Indication for systemic anti-HER2 treatment<br>• Prior exposure to trastuzumab and pertuzumab<br>• Prior exposure to T-DM1 allowed<br>• Life expectancy of at least 3 months<br>• Age ≥18 years<br>• Patient must be able to tolerate therapy, and have adequate cardiac function (defined by baseline left ventricular ejection fraction ≥50%)<br>• Adequate bone-marrow, liver and kidney function<br>• Adequate treatment washout period before enrolment, defined as:<br>• Major Surgery: ≥4 weeks<br>• Radiation therapy: ≥4 weeks<br>• Chemotherapy, small-molecule targeted agents, anticancer hormonal therapy: ≥3 weeks<br>• Antibody-based treatment: ≥4 weeks<br>• Patient must be capable of understanding the purpose of the study and have given written informed consent

EXCLUSION CRITERIA	• Metastatic breast cancer other than HER2-positve disease<br>• Use of any investigational agent within 28 days prior to initiation of treatment<br>• History of malignancy other than squamous cell carcinoma, basal cell carcinoma of the skin or carcinoma in situ of the cervix within the last 3 years including contralateral breast cancer<br>• Major surgery, other than diagnostic surgery, within the last 4 weeks<br>• Indication for immediate local therapy as defined by local standard<br>• Leptomeningeal involvement<br>• Other anticancer therapy, including cytotoxic, targeted agents, immunotherapy, antibody, retinoid, or anti-cancer hormonal treatment<br>• Concomitant radiotherapy<br>• Prior radiotherapy to the thorax other than breast irradiation or irradiation of bone metastases<br>• A history of uncontrolled seizures, central nervous system disorders or psychiatric disability judged by the investigator to be clinically significant and adversely affecting compliance to study drugs<br>• Clinically significant cardiac disease including unstable angina, acute myocardial infarction within six months prior to randomization, congestive heart failure (NYHA III-IV), left ventricular ejection fraction <50%, arrhythmia unless controlled by therapy, with the exception of extra systoles or minor conduction abnormalities, and long QT syndrome (QTc interval >450 ms)<br>• Subjects who have current active hepatic or biliary disease (with exception of patients with Gilbert's syndrome, asymptomatic gallstones, liver metastases or stable chronic liver disease per investigator assessment) including acute and chronic infections with hepatitis B and C<br>• Inadequate haematological status at baseline prior to study entry: Dependency on red blood cell and/or platelet transfusions, ANC (absolute neutrophil count (segmented + bands) <1.0 x 10^9/L; platelets <100 x 10^9/L<br>• Inadequate kidney function: serum-creatinine >1.5 times upper normal limit

	• Hepatic dysfunction: total bilirubin >1.5 times upper normal limit (>3 in patients with liver metastases or known history of Gilbert's disease); ALT, AST >3 times upper normal limit (>5 in patients with liver metastases); serum albumin <2.5 g/dL; INR ≥1.5<br>• Clinically severe pulmonary compromise resulting from intercurrent pulmonary illnesses including, but not limited to, any underlying pulmonary disorder (i.e. pulmonary emboli within three months of the study enrolment, severe asthma, severe COPD, restrictive lung disease, pleural effusion etc.), and any autoimmune, connective tissue or inflammatory disorders with pulmonary involvement (i.e. rheumatoid arthritis, Sjogren's syndrome, sarcoidosis etc.), or prior pneumonectomy.<br>• Subjects with bronchopulmonary disorders who require intermittent use of bronchodilators (such as albuterol) will not be excluded from this study<br>• Patients with active opportunistic infections<br>• Known HIV infection<br>• Concomitant treatment with chloroquine or hydroxychloroquine<br>• Pregnant or lactating women. Women with childbearing potential must have a negative pregnancy test at screening<br>• Women with childbearing potential, including women whose last menstrual period was less than one year prior to screening, unable or unwilling to use adequate contraception from study start to one year after the last dose of protocol therapy. Acceptable contraception methods included the application of an intrauterine device, barrier method or total abstinence<br>• Patients with known hypersensitivity to trastuzumab<br>• Patients not able to provide written informed consent<br>• Patients with known substance abuse or any other medical conditions such as clinically significant cardiac or psychological conditions, that may, in the opinion of the investigator, interfere with the subject's participation in the clinical study or evaluation of the clinical study results<br>• Patients requiring concomitant use of chronic systemic (IV or oral) corticosteroids at doses higher than 4 mg dexamethasone per day or other immunosuppressive

	medications except for managing adverse events (inhaled steroids or intra articular steroid injections are permitted in this study)

PLANNED STUDY DURATION	Stage 1: Enrolment period 8 months<br>Stage 2: Enrolment period 12 months<br>FPFV: July 2020, LPLV August 2022
TEST DRUG (IMP)	Trastuzumab-deruxtecan 5.4 mg/kg body weight i.v. on day 1 once every three weeks
CONCOMITANT MEDICATION	Supportive concomitant medication is allowed according to local treatment standard. A detailed list of forbidden concomitant medication is provided in section 8.5.2
EFFICACY ENDPOINTS	• Primary endpoint: Response rate (CNS) at any timepoint as judged by best response during the study period (clinical response measured according to RANO-BM criteria)<br>• Secondary endpoints: Clinical benefit rate (CNS defined by RANO-BM; CR+PR+SD ≥6 months), extracranial response rate and clinical benefit rate (RECIST 1.1), progression-free survival, time-to-WBRT, overall survival
TOLERABILITY / SAFETY ENDPOINTS	• Endpoint: Safety & tolerability of trastuzumab-deruxtecan in terms of haematologic and non-haematologic side effects as assessed by the investigators<br>• Assessment of clinical adverse events & laboratory parameters
QUALITY OF LIFE ENDPOINTS	• Endpoint: Evaluation of Quality-of-Life (QoL) in patients receiving trastuzumab-deruxtecan for HER2-positive breast cancer brain metastases<br>• Assessment of QoL with EORTC QLQ-c30, the brain specific tool (BN20), and the breast specific tool BR45
EXPLORATORY ENDPOINTS	• Endpoint: Evaluation of biomarkers potentially predicting for response to trastuzumab-deruxtecan<br>• Assessment of HER2 gene copy numbers and the rate of tumour-infiltrating lymphocytes in primary tumour or (preferentially) from a metastatic biopsy<br>• Blood samples for ancillary studies at baseline, before cycle 4 and at EOT
STATISTICAL CONSIDERATIONS	**Efficacy:** Primary endpoint is the rate of objective responses at any timepoint as judged by best response during therapy, i.e. complete remission (CR), partial

remission (PR), stable disease (SD) / no change and progressive disease as defined by RANO response criteria

This is a single-arm non-comparative phase II study evaluating the rate of objective CNS responses to trastuzumab-deruxtecan in HER2-positive metastatic breast cancer patients with brain metastases.

A Simon's two-stage phase II design will be applied. A response rate of 61% would be considered as indicating clinically relevant activity in this patient population. A response rate of <26%, on the other hand, must be considered clinically unmeaningful (null hypothesis).

In the first stage, 6 patients will initially be accrued. If there are two or fewer responses in these 6 patients, the study will be stopped. Otherwise, 9 additional patients will be accrued for a total number of 15 patients. The null hypothesis will be rejected if 7 or more responses are observed in these 15 patients. This design yields a type I error rate of 5% and a power of 80% to reject the null-hypothesis.

P0=0.250, P1=0.610, Alpha=0.050, Beta=0.200

**N1**	**R1**	**N**	**R**	**Alpha**	**Beta**
6	2	15	6	0.050	0.200

N1 is the sample size in the first stage.

R1 is the drug rejection number in the first stage.

N is the combined sample size of both stages.

R is the combined drug rejection number after both stages.

Alpha is the probability of rejecting that P<=P0 when this is true.

Beta is the probability of rejecting that P>=P1 when this is true.

P0 is the response proportion of a poor drug.

P1 is the response proportion of a good drug.

<u>Tolerability and Safety:</u>

	Endpoints are safety & tolerance of treatment in terms of haematologic and non-haematologic side effects as assessed by the investigators. Adverse events will also be summarised using frequency counts and percentages.<br><br>All patients who are eligible for the study and receive at least one dose of study drug l be will be included in the safety analysis.<br><br>Patients who drop out or die prior to the first response assessment will be included in the denominator when calculating the response rate. Any patients who enrol into the study but who receive no study medication will be excluded, but a sensitivity analysis will be conducted which will include all enrolled patients. An analysis of all patients undergoing treatment per protocol excluding drop out will also be performed.

LIST OF ABBREVATIONS

ADC	Antibody-Drug Conjugate
ADR	Adverse Drug Reaction
AE	Adverse Event
AESI	Adverse Event of Special Interest
ALT	Alanine Aminotransferase
AST	Aspartate Aminotransferase
BC	Breast Caner
BM	Brain Metastases
CBR	Clinical Benefit Rate
CR	Complete Remission
CRF	Case Report Form
CRO	Clinical Research Organization
CSR	Clinical Study Report
CT	Computed Tomography
DOH	Declaration of Helsinki
DSUR	Development Safety Update Report
ECG	Electrocardiography
ECOG	Eastern Cooperative Oncology Group
EORTC	European Organisation for Research and Treatment of Cancer
EOS	End of Study
EU	European Union
FPFV	First Patient First Visit

GCP	Good Clinical Practice
GGT	Gamma-Glutamyltransferase
HBsAg	Hepatitis B Surface Antigen
HBV	Hepatitis B Virus
HCG	Human Corionic Gonadotropin
HER2	Human Epidermal Growth Factor Receptor 2
HVC	Hepatitis C Virus
HIV	Human Immunodeficiency Virus
IB	Investigator's Brochure
ICH	International Conference on Harmonization
IEC	Independent Ethics Committee
IMP	Investigational Medicinal Product
ISF	Investigator Site File
ILD	Interstitial Lung Disease
ISO	International Standardisation Organization
KKS	Koordinationszentrum für Klinische Studien
LPLV	Last Patient Last Visit
LVEF	Left Ventricular Ejection Fraction
mL	Milliliter
mm	Millimeter
MRI	Magnetic Resonance Imaging
MUGA scan	Multigated Acquisition Scan
MUW	Medical University of Vienna
NCI CTCAE	National Cancer Institute Common Terminology Criteria for Adverse Events
OS	Overall Survival

PARP	Poly (ADP-ribose) Polymerase
PFS	Progression-free Survival
PD-L1	Programmed cell Death Ligand 1
PK	Pharmacokinetics
PR	Partial Remission
PD	Progressive Disease
QoL	Quality-of-Life
RANO	Response Assessment in Neuro-Oncology
RBC	Red Blood Cells
RR	response Rate
SAE	Serious Adverse Event
SAP	Statistical Analysis Plan
SAR	Serious Adverse Reaction
SD	Stable Disease
SmPC	Summary of Product Characteristics
SOP	Standard Operating Procedure
STX	Stereotactic Radiotherapy
SUSAR	Suspected Unexpected Serious Adverse Reaction
T-DM1	Trastuzumab-DM1
T-DXd	Trastuzumab-Deruxtecan
TEAE	Treatment-emerging adverse event
TILs	Tumour-Infiltrating Lymphocytes
TMF	Trial Master File
TKI	Tyrosine-Kinase Inhibitor
WBC	White Blood Cells

WBRT	Whole Brain Radiotherapy
WHO	World Health Organization

TABLE OF CONTENTS

PROTOCOL SYNOPSIS **4**

1. BACKGROUND **24**

1.1 BREAST CANCER BRAIN METASTASES 24
1.2 TRASTUZUMAB-DERUXTECAN BACKGROUND AND RATIONAL 27

2. STUDY DESIGN **32**

2.1 STUDY OBJECTIVES 32

3. STUDY DURATION **33**

4. NUMBER OF CENTRES **34**

5. SELECTION CRITERIA **34**

5.1. TOTAL NUMBER OF PATIENTS 34
5.2. INCLUSION CRITERIA 34
5.3. EXCLUSION CRITERIA 35

6. DISEASE EVALUATION **37**

6.1 PRIMARY ACTIVITY PARAMETERS 37

7. STUDY MEDICATION **40**

7.1 TRASTUZUMAB-DERUXTECAN 40

8. SCREENING, ELIGIBILITY & TREATMENT **42**

8.1 STUDY TREATMENT 42
8.2 STUDY PROCEDURES 42
8.3 DOSE CONTINUATION, MODIFICATION & INTERRUPTION 43
8.4 TREATMENT COMPLIANCE 54
8.5 CONCOMITANT THERAPY 54

9. PREMATURE WITHDRAWAL **55**

9.1 PREMATURE TRIAL TERMINATION 55
9.2 REPLACEMENT POLICY 55

10. STATISTICAL CONSIDERATIONS **55**

10.1 SAMPLE SIZE CALCULATION 55
10.2 EFFICACY 57
10.3 TOLERABILITY & SAFETY 58
10.4 QUALITY-OF-LIFE 58
10.5 EXPLORATORY ENDPOINTS 58
10.6 ANALYSIS PLAN 58
10.7 RELEVANT PROTOCOL DEVIATIONS 58

10.8 MISSING, UNUSED AND SPURIOUS DATA 58

11. SAFETY 59

11.1 ADVERSE EVENTS 59
11.2 SERIOUS ADVERSE EVENTS 59
11.3 ADVERSE EVENT OF SPECIAL INTEREST 60
11.4 CLASSIFICATION OF SEVERITY 61
11.5 CLASSIFICATION OF RELATIONSHIP/CAUSALITY OF ADVERSE EVENTS (AE/SAE) TO IMP .. 62
11.6 SAE AND SUSAR REPORTING 62
11.7 PREGNANCIES 64

12. QUALITY CONTROL & QUALITY ASSURANCE 65

12.1 PERIODIC MONITORING 65
12.2 AUDITS & INSPECTIONS 65
12.3 PUBLICATION OF STUDY RESULTS 65

13. ETHICAL & LEGAL ASPECT 65

13.1 INFORMED CONSENT OF SUBJECTS 65
13.2 ACKNOWLEDGEMENT/APPROVAL OF THE STUDY 66
13.3 PROTOCOL AMENDMENTS 66
13.4 STUDY TERMINATION 66
13.5 CLINICAL STUDY REPORT (CSR) 67
13.6 FINANCE & INSURANCE 67
13.7 ETHICS AND GOOD CLINICAL PRACTICE (GCP) 67

14. APPENDICES 68

FIGURE 1 STUDY FLOW

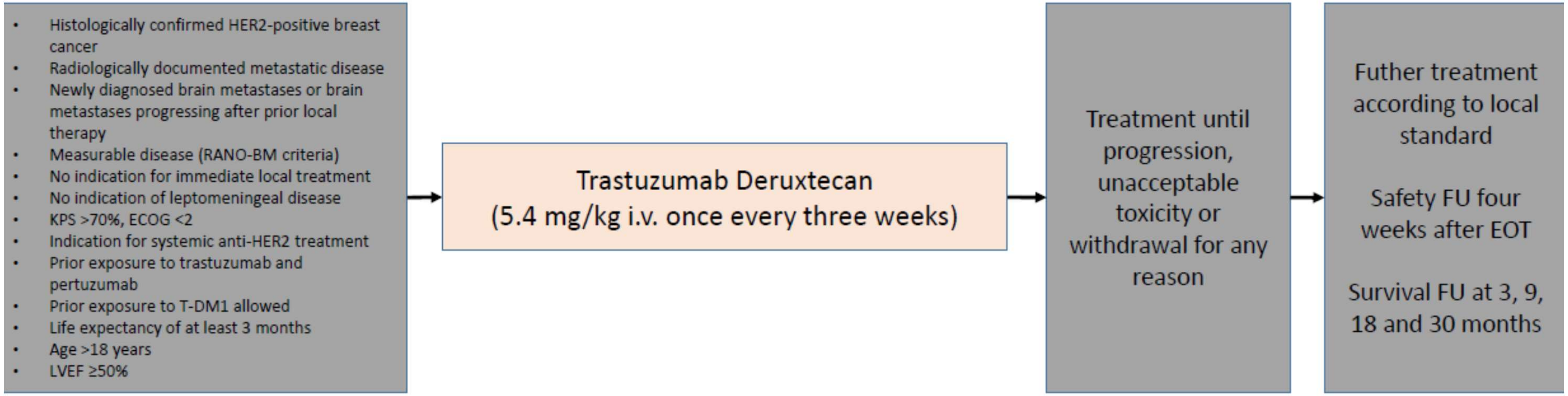

TABLE 1 VISIT AND ASSESSMENT SCHEDULE

	SCR	*Cycle 1*				*Cycle 2*		*Cycle 3*		*Cycle 4 and subsequent cycles Day 1*		*EOT*	*Safety F/U (4 weeks after EOT)*	*OS F/U at 3, 9, 18 and 30 m*
		Day 1		*Day 8*	*Day 15*	*Day 1*		*Day 1*						
	-28 days	*BI[(a)]*	*EOI*	*(±1 day)*	*(±1 day)*	*BI[(a)]*	~~*EOI*~~	*BI[(a)]*	~~*EOI*~~	*BI[(a)]*	~~*E OI*~~			
Medical history	●													
Demographic	●													
HIV Antibody Test (b)	●													
Hepatitis B/C serology (b)	●													
Pregnancy Test (c)	●											●		
Vital Signs	●	●	●	●	●	●	~~●~~	●	~~●~~	●		●	●	
Physical Examination	●	●				●		●		●		●	●	
SpO_2		●				●		●		●				
Height	●													
Weight, ECOG PS	●	●				●		●		●		●	●	

	SCR	Cycle 1				Cycle 2		Cycle 3		Cycle 4 and subsequent cycles Day 1		EOT	Safety F/U (4 weeks after EOT)	OS F/U at 3, 9, 18 and 30 m
		Day 1		Day 8	Day 15	Day 1		Day 1						
	-28 days	BI(a)	EOI	(±1 day)	(±1 day)	BI(a)	~~EOI~~	BI(a)	~~EOI~~	BI(a)	~~E OI~~			
Hematology & Blood Chemistry Tests (d)	●	●		●	●	●		●		●		●	●	
Troponin (ed)	●											●		
ECHO or MUGA (LVEF) (fe)	●(bf)									●		●		
12-lead ECG in triplicate (g)	●	●										●		
CT-chest/abdomen (h)	●							●(i)		Before cycle 5 and every 9 weeks thereafter (i,j)				
Cranial MRI	●							● (i)		Before cycle 5 and every 9 weeks thereafter (i,j)				
Quality-of-Life		●						●		Before cycle 5 and every 9 weeks thereafter		●		Final QoL analy sis at 3 mont hs

	SCR	*Cycle 1*				*Cycle 2*		*Cycle 3*		*Cycle 4 and subsequent cycles Day 1*		*EOT*	*Safety F/U (4 weeks after EOT)*	*OS F/U at 3, 9, 18 and 30 m*
		Day 1		*Day 8*	*Day 15*	*Day 1*		*Day 1*						
	-28 days	*BI[(a)]*	*EOI*	*(±1 day)*	*(±1 day)*	*BI[(a)]*	~~*EOI*~~	*BI[(a)]*	~~*EOI*~~	*BI[(a)]*	~~*EOI*~~			
Bone Scan (h)	•									*Whenever clinically indicated*				
Ophthalmologic Assessments (k)	•*(b)*											•		
Urinalysis	•													
Tissue Sample for Biomarker Studies (l)	•													
Blood Samples for Ancillary Studies (optional)		•								•		•		
Concomitant Medications	•													
AEs	•													
Survival F/U														•

a Within 3 days before administration
b Within 28 days before randomization/enrollment
c Within 72 hours before enrollment for all female subjects of childbearing potential; a positive urine pregnancy test result must immediately be confirmed using a serum test. Perform repeat pregnancy tests (urine or serum test per institutional guideline) 72 hours before infusion of each cycle and at end of treatment.
d Laboratory tests include: Hematology - red blood cell count, hemoglobin, hematocrit, platelet count, white blood cell count, differential white blood cell count (neutrophils, lymphocytes, monocytes, eosinophils, basophils) and Chemistry - total protein, albumin, ALP, ALT, AST, total bilirubin, BUN, Ca, Cl, serum creatinine, LDH, K, Na and magnesium

e Collect blood samples for troponin (preferably high-sensitivity troponin-T) at screening, EOT, and if at any time a subject reports signs or symptoms suggesting congestive heart failure, myocardial infarction, or other causes of myocyte necrosis.
f ECHO or MUGA scan assessments (note: the same test must be used for the subject throughout the study) will be performed at Screening and BI on Day 1 of Cycle 5 and then every 4 cycles (±7 days) (e.g. Cycle 5, 9, 13…)
g ECGs will be taken in close succession, while in a supine/semi-recumbent position
h With further workup as indicated
i Within 10 days before the next treatment cycle
j Staging investigations should be conducted whenever disease progression is suspected
k Ophthalmologic assessments including visual acuity testing, slit lamp examination and fundoscopy will be performed at screening and EOT
l Tissue should be collected from the primary tumour or (preferentially)from a metastatic biopsy

For suspected ILD/pneumonitis, study drug should be interrupted pending evaluation, which should include:

- high resolution CT
- pulmonary consultation (Infectious Disease consultation as clinically indicated)
- Blood culture and CBC. Other BLOOD tests could be considered as needed
- Consider bronchoscopy and bronchoalveolar lavage if clinically indicated and feasible
- pulmonary function tests and pulse oximetry (SpO2)
- arterial blood gases if clinically indicated
- one blood sample collection for PK analysis as soon as ILD/pneumonitis is suspected, if feasible

Other tests could be considered as needed.

1. BACKGROUND

1.1 BREAST CANCER BRAIN METASTASES

Up to 15% of all patients with metastatic breast cancer (MBC) will develop brain metastases (BM) during their course of disease, making MBC after lung cancer the second most common cause of BM among solid malignancies [1]. Incidence of MBC BM has been rising over the last years commonly attributed to the tremendous progress of systemic therapy resulting in prolonged overall survival (OS) of MBC patients with human epidermal growth factor receptor 2 (HER2) positive disease [2] and a hypothetical shift to more aggressive courses of MBC in patients recurring after optimal adjuvant treatment [3]. Screening of asymptomatic patients and in consequence the more frequent diagnosis of a- to oligosymptomatic patients might be further attributing to the increasing incidence of breast cancer (BC) BM patients. Prognosis of patients with BM remains poor with median OS times ranging from 2 to 16 months and differs greatly depending on the BC subtype [1].

In daily clinical practice, BC subtypes are classified as luminal, triple-negative, and HER2-positive as defined by the expression of the oestrogen- (ER) and/or progesterone-receptor (PR) and HER2 overexpression as defined by immunohistochemistry and/or *HER2/neu* gene amplification [4 ,5]. Treatment of different BC subtypes differs substantially since endocrine therapy (ET) is the standard-of-care for luminal disease and HER2-targeted treatment plays a pivotal role in HER2-positive BC. Chemotherapy remains the backbone of treatment for triple-negative breast cancer (TNBC) patients with inhibitors of PARP and PD-L1 just recently introduced. Patients with triple-negative tumours are at greatest risk for being diagnosed with BM followed by patients with HER2-positive disease [6]. In addition to BM incidence, brain metastases free survival (BMFS) was shown to differ between subtypes as well [7].

Treatment decisions in BM patients should be based on clinical characteristics such as Karnofsky performance score, age, number of BM and the status of extracranial disease [8]. Local therapy (surgical resection, stereotactic radiosurgery (SRS) und whole brain radiotherapy (WBRT)) remains the mainstay of treatment, especially for symptomatic patients with a need for rapid symptom relief [8,9]. By contrast, about 20% of BC patients are diagnosed with asymptomatic BM which may have a better prognosis [10]. For these patients, systemic treatment has become an attractive alternative approach since long-term toxicity of WBRT can be postponed. In certain

areas of BM, the blood-brain barrier (BBB) is disrupted and replaced by a blood-tumour barrier with higher fenestration of the endothelium allowing bigger molecules to penetrate the brain parenchyma [11,12].

1.1.1. Lapatinib as upfront systemic therapy for BM

The prospective non-comparative single-arm phase II LANDSCAPE trial accrued 45 patients with HER2-positive BC and newly diagnosed BM; more than 40% of all patients had no neurological symptoms at diagnosis, therefore representing an asymptomatic screening population [13]. CNS response rate was specified as the primary study endpoint and defined as a 50% or greater volumetric reduction of CNS lesions in the absence of increased steroid use, progressive neurological symptoms, or progressive extra-cranial disease. At a median follow-up of 21.2 months, CNS response rate in this selected population was 65.9% (95% CI 50.1-79.5) and PFS 5.5 months (95% CI 4,3-6). Other relevant secondary endpoints included time-to-WBRT (8.3 months; 95% CI 5.4-9.1) and OS (17.0 month; 95%CI 13.7-24.9). On the downside, 49% of all study subjects experienced grade 3/4 adverse events indicative of relevant toxicity while no quality-of-life data are available. Despite this, LANDSCAPE defined a potential new treatment standard for patients with multiple BM without severe neurological symptoms.

1.1.2. Neratinib and Tucatinib in BM

Neratinib is a second generation (irreversible) TKI of HER2 and EGFR. The direct activity of neratinib in HER2-positive BC BM was evaluated in a multicohort prospective phase II trial (TBCRC022) including forty patients whose BM had progressed on prior local therapy (78% WBRT) [14]. In this study, however, activity of single agent neratinib was disappointing with a CNS response rate of 8%; median PFS was 1.9 months. Another cohort of this phase II study evaluated the combination of neratinib and capecitabine (with upfront loperamide prophylaxis) [15]. Thirty-seven patients with progressive BM were included. A CNS response rate of 49% (95% CI 32-66%) was reported; PFS and OS were 5.5 and 13.5 months, respectively, indicating relevant clinical activity. On the downside, grade 3 diarrhoea was observed in 32% of patients despite prophylaxis.

Tucatinib (ONT-380) is a third generation HER2 TKI. In contrast to lapatinib and neratinib, this drug has only minor inhibitory activity against EGFR resulting in a lower diarrhoea-rate [16]. In a joint analysis of two phase Ib studies investigating different tucatinib-based combinations, the rate of patients with prolonged PFS (defined as PFS ≥16 months) was evaluated [17]. Overall, 22% of this

heavily pretreated population achieved prolonged disease control. Of note, 50% of these patients had BM at baseline indicating a promising activity of tucatinib in this patient subset.

The prospective randomized phase II HER2CLIMB randomized 612 HER2-positive MBC patients to the triple combination of trastuzumab, capecitabine and tucatinib or trastuzumab, capecitabine and placebo [18]. Nearly half of these patients had a known history of brain metastases at baseline; importantly, approximately 40% of these had active (i.e. previously untreated or progressing) BM. In this heavily pretreated population (prior treatment with trastuzumab, pertuzumab and T-DM1 was required), a significant prolongation of progression-free survival (HR 0.54; 95% CI 0.42-0.71; *p*=0.00001) and overall survival ()HR 0.66; 95% CI 0.5-0.88; *p*=0.0048) was observed in favour of the tucatinib group. In the subset of BM patients, a similar benefit in terms of PFS was reported (HR 0.48; 95% CI 0.34-069; *p*<0.00001) with 25% of patients free of progression at one year *versus* 0%. CNS response rates in patients with active BM, however, are still awaited.

1.1.3. Trastuzumab-DM1 in BM

Trastuzumab passes an impaired BBB at the site of BM while similar to lapatinib, no significant uptake of radioactively tagged trastuzumab in healthy brain tissue was observed [19]. In addition, there is currently no prove of direct activity of trastuzumab monotherapy in newly diagnosed or progressing BM. This led to growing interest regarding the potential activity of trastuzumab-DM1 (T-DM1), an antibody-drug-conjugate linking the anti-microtubule-agent DM1 to trastuzumab.

The phase III EMILIA trial compared T-DM1 with lapatinib plus capecitabine in the second-line setting and in first-line patients progressing on adjuvant trastuzumab or within six months since the end of adjuvant immunotherapy; inclusion of patients with stable BM after local therapy was allowed [20]. In the overall population, T-DM1 was associated with a significant and clinically relevant advantage over lapatinib plus capecitabine in terms of PFS (9.6 *vs*. 6.4 months; HR 0.65; 95% CI 0.55-0.77; *p*<0.001) and OS (30.9 *vs*. 25.1 months; HR 0.68; 95% CI 0.55-0.85; *p*<0.001). In patients with stable BM at baseline (*n*=95), superiority of T-DM1 was maintained although not surprisingly, OS in absolute numbers was shorter in both arms (26.8 *vs*. 12.0 months; HR 0.38; *p*=0.008) [21].

Meanwhile, several reports and case series suggested clinically relevant activity of T-DM1 in newly diagnosed or progressive BM. In a population of ten patients with HER2-positive BM, T-DM1 was

administered as upfront therapy for newly diagnosed BM (n=2) or upon progression of BM after prior local therapy (n=8) [22]. Thus, this analysis allowed for an appraisal of the direct activity of T-DM1 in BM. A partial remission as defined by RANO BM response criteria (decrease in the sum of longest diameters of CNS target lesions of at least 30% sustained for at least 4 weeks in the absence of new lesions, increased corticosteroid dose and/or clinical deterioration [23]) was observed in three patients (PR, 30%), while disease stabilization of ≥6 months was reported in two additional subjects, resulting in a clinical benefit rate (CR, PR, SD ≥6 months) of 50%. In addition, two further patients experienced stable disease ≥3 months but <6 months. Of note, activity was not restricted to patients with newly diagnosed BM as one of the two responders and both patients with SD ≥6 months had progressive BM at baseline. At a median follow-up of 8.5 months, intracranial PFS was 5 months (95% CI 3.69-6.32), and median OS from initiation of T-DM1 based treatment had not been reached. Another retrospective study included thirty-nine patients from five French centres [24]. Patients had received a median of two prior HER2-directed treatment lines for metastatic disease and 36 patients had received prior local therapy as well, consisting mostly of WBRT (72%). In this pretreated population, a response rate of 44 % was reported; median PFS was 6.1 months (95 %CI 5.2–18.3). These clinical data are supported by a preclinical model of trastuzumab or T-DM1 at equivalent doses in female nude mice with BM [25]. Median survival in mice bearing BM generated with the luminal B/HER2-positive BT474 cell line was 28 days for trastuzumab and 112 days for T-DM1 (HR 6.2; 95% 6.1-85.84; p<0.001). In addition, a significantly higher rate of tumour cell apoptosis was observed in the T-DM1 group.

In summary, these clinical and preclinical data suggest that T-DM1 harbours clinically relevant activity in MBC BM; the prospective phase II KIARA (Kadcyla In pAtients With bRAin Metastasis) trial (NCT03203616) intended as prospective verification of this concept, however, was recently stopped due to poor accrual. Concurrent administration of T-DM1 and radiosurgery, on the other hand, may not be advisable as a rate of irradiation necrosis of 50% was observed in another small retrospective case series [26].

1.2 TRASTUZUMAB-DERUXTECAN BACKGROUND AND RATIONAL

1.2.1. Trastuzumab-Deruxtecan

Trastuzumab-deruxtecan (T-DXd) is a antibody-drug conjugate consisting of a humanized HER2-directed monoclonal antibody (MAAL-9001) with the same amino acid sequence as trastuzumab

and the cytotxocic agent deruxtecan (MAAA-1181a), a potent inhibitor of topoisomerase I with a drug to antibody ratio of 8 [27].

- Preclinical Data

Results of *in vitro* cell growth inhibition studies conducted using several cancer cell lines have shown that T-DXd has a more potent growth inhibitory effect against HER2-positive cells than MAAL-9001 does, suggesting that the conjugation of MAAA-1181a enhances the growth inhibition of T-DXd. Moreover, no growth inhibition was observed in HER2-negative cells, confirming the HER2 specificity of T-DXd. Similarly, when the in vivo antitumor activity of T-DXd in a tumour-bearing mouse model of a HER2-positive gastric cancer cell line (NCI-N87) was studied, it was confirmed that the drug exhibited potent, dose-dependent antitumor activity with tumour regression and that this activity was even stronger than that of MAAL-9001. T-DXd also exhibited antitumor activity in a tumour-bearing mouse model of human breast cancer cell line (KPL-4). Of note, T-DXd demonstrated more potent antitumor activity than T-DM1 in several mouse xenograft models of breast and gastric cancer.

Information regarding safety pharmacology, pharmacokinetics and toxicology are provided in the IB [27].

- Clinical Data: Clinical Pharmacology

Preliminary PK data from the Dose Escalation part of the DS8201-A-J101 study showed that the Cmax of T-DXd was dose proportional across the dose range of 0.8 mg/kg to 8.0 mg/kg. The area under the serum concentration-time curve up to the last quantifiable time (AUClast) increased greater than dose proportionally from 0.8 mg/kg to 3.2 mg/kg and then increased dose proportionally from 3.2 mg/kg and above. On a molar basis, T-DXd exposures at the 5.4-mg/kg dose were approximately 39-fold to 44-fold higher than those for MAAA-1181a. Preliminary PK data from the Dose Expansion part showed that the T-DXd, total anti-HER2 antibody, and MAAA-1181a exposures were similar between HER2-positive and HER2-low breast cancer. The T-DXd and total anti-HER2 antibody exposures appeared to be numerically lower in HER2-positive gastric/gastroesophageal junction (GEJ) cancer than in breast cancer. MAAA-1181a exposures were consistent across all tumour types evaluated.

Preliminary PK data of the DS8201-A-U201 study showed that the ratio of T-DXd and total anti-HER2 antibody was approximately 1 for Cmax and approximately 0.8 for AUC and trough concentrations. The serum concentrations of MAAA-1181a gradually increased and reached peak concentrations, with longer median Tmax (approximately 7 hours) than those for T-DXd (approximately 4 hours). On a molar basis, the T-DXd Cmax and trough concentrations were approximately 54-fold and 74-fold higher, respectively, than those for MAAA-1181a. These results demonstrate the in vivo stability of the ADC.

In the DS8201-A-J102 study, the concentration profile of total anti-HER2 antibody was similar to that of T-DXd. The exposure of MAAA-1181a was low compared with those of T-DXd and total anti-HER2 antibody. The mean accumulation ratio (AR) for area under the plasma concentration-time curve during dosing interval (AUCtau) at Cycle 3 was 1.35 for T-DXd, 1.36 for total anti-HER2 antibody, and 1.09 for MAAA-1181a.

The concentration profile of total anti-HER2 antibody was similar to that of T-DXd in study DS8201-A-A103. Some accumulation (approximately 57%) of T-DXd was observed at Cycle 3. The systemic exposure to MAAA-1181a was lower than that of T-DXd, but t1/2 values were similar across the 3 analytes.

Final PK data from DS8201-A-A104 showed that concomitant use of ritonavir or itraconazole resulted in minimal increases in exposure of MAAA-1181a, with increases in area under the concentration-time curve up to 17 days (AUC17d) of 22% and 18%, respectively. Concomitant use of ritonavir or itraconazole resulted in minimal increases in exposure of T-DXd, with increases in AUC17d of 19% and 11%, respectively. The results indicate that concomitant use of ritonavir (dual inhibitor of OATP1B/CYP3A) or itraconazole (strong CYP3A inhibitor) would not result in clinically meaningful increases in exposures of T-DXd or MAAA-1181a.

- Clinical Data: Efficacy

DS8201-A-J101

Preliminary efficacy results of this phase I trial showed confirmed objective RR (ORR) by independent central review (ICR) of 52.5% (95% confidence interval [CI]: 43.1, 62.0) among the 118 subjects with HER2-positive breast cancer. RR was 51.0% (95% CI 36.6-65.2) in the 5.4-mg/kg dose group and 53.7% (95% CI 41.1-66.0) in the 6.4-mg/kg dose group, respectively. The median

confirmed duration of response was 13.3 months and median PFS 13.7 months; median OS was not reached as of the data cut-off.

Among the 54 subjects with HER2-low breast cancer, confirmed objective RR was 37.0% (95% CI 24.3-51.3). A median PFS of 11.1 months (95% CI 7.6-not estimable) and median OS was 29.4 months (95% CI 12.9-29.4) war reported. Among the 44 subjects with HER2-positive gastric/GEJ cancer, confirmed objective RR was 29.5% (95% CI 16.8-45.2); median PFS was 5.4 months (95% CI 4.1-8.5) and median OS 20.2 months (95% CI 10.0-not estimable), respectively. Finally, in the group of 61 patients with other cancers, confirmed RR was 29.5% (95% CI 18.5-42.6). Median PFS was 7.2 months (95% CI 4.8-11.1) and median OS was 23.4 months (95% CI 15.6-not estimable).

DESTINY-Breast01

In the single-arm phase II trial DESTINY-Breast01, 253 pretreated HER2-positive MBC patients received T-DXd at different doses [28]. After PK and dose-finding stage, 5.4 mg/kg body once every three weeks weight was defined as the recommended dose for further development. All patients had received prior treatment with trastuzumab and T-DM1. In addition, two thirds had received prior pertuzumab and 54% other HER2-targeted agents as well; the median number of prior treatment lines was 6 (range 2-27). Efficacy analysis included 184 patients treated with 5.4 mg/kg. In this heavily pretreated population, a clinically relevant response rate of 60.9% was observed; median PFS was 16.4 months (95% CI 12.7-NE) and median OS not yet reached. In 24 patients with stable brain metastases at baseline, activity was comparable (median PFS 18,1 months; 95% CI 6.7-18.1 months). Based upon these results, no final conclusion regarding the activity T-DXd in active BM can be drawn; however, these data indicate that further investigation of this drug in breast cancer brain metastases is warranted.

- Clinical Data: Safety

As of June 8th 2019, based on the cumulative review of the safety data, including available nonclinical, clinical, and epidemiologic information and scientific literature (published and unpublished) and taking into consideration biological plausibility, interstitial lung disease (ILD), anaemia, neutrophil count decrease including febrile neutropenia, and platelet count decrease are classified as important identified risks. Left ventricular ejection fraction (LVEF) decrease is classified as an important potential risk. Infusion-related reactions, which were previously classified as an important potential risk, are reclassified as an identified risk. QT prolongation is

no longer considered an important potential risk and has been removed from the list of safety concerns for T-DXd. Both QT prolongation and infusion-related reactions are no longer considered adverse events of special interest (AESIs).

In the T-DXd clinical program, the inclusion/exclusion criteria and monitoring/management guidelines are currently in place in all protocols to mitigate the important identified risks of ILD, anaemia, neutrophil count decrease including febrile neutropenia, and platelet count decrease, and important potential risk of LVEF decrease.

Interstitial lung disease is a known serious risk of T-DXd, and cases with fatal outcomes have been reported. Most events were grade 1 or grade 2 and were manageable by dose modification and following clinical treatment guidelines for drug-induced ILD, with specific recommendations including close monitoring of signs/symptoms of ILD (e.g. cough, fever, and dyspnoea) to identify potential ILD and proactively managing ILD with dose modification, treatment interruption and application of immunosuppressive drugs

Other identified risks of trastuzumab deruxtecan in order of descending frequencies are nausea, decreased appetite, alopecia, vomiting, fatigue, constipation, diarrhoea, white blood cell (WBC) count decrease, stomatitis, aspartate aminotransferase increased, cough, headache, abdominal pain, alanine aminotransferase increased, hypokalaemia, epistaxis, dyspnoea, dyspepsia, dizziness, dry eye, upper respiratory tract infection, asthenia, and infusion-related reactions. These identified risks were generally manageable through dose modification and routine clinical practice.

1.2.2. Rationale for Performing the Study

The conventional standard treatment of multiple BM is WBRT. With highly active HER2-directed drugs available today, prolonged survival >24 months despite the presence of BM is possible; such patients are therefore at risk of experiencing WBRT-associated neurological decline. TKIs such as lapatinib and neratinib and the ADC T-DM1 were shown to exert clinically relevant activity in both newly diagnosed BM and patients with BM progressing after prior local treatment. The combination of lapatinib or neratinib with capecitabine, however, is associated with a high rate of severe diarrhoea, thereby hampering the broad clinical use of these combination. In addition, T-DM1 is currently the standard-of-care in the second-line setting and also used as first-line therapy in patients with early progression on adjuvant trastuzumab when BM may not yet be

present; increasingly T-DM1 is also being used in the postneoadjuvant setting based upon the KATHERINE trial [29]. These facts render the development of novel active and well-tolerated drugs in the setting of HER2-positive BC patients with BM necessary.

As outlined above, T-DXd is a highly active HER2-directed ADC showing relevant clinical activity even after progression on T-DM1 with a favourable safety profile. As the rate of patients with early T-DM1 exposure is expected to increase further in the future, T-DXd appears to be an attractive candidate for the evaluation in pretreated patients with brain metastases. Of note, results of the phase II DESTINY-Breast01 trial suggested that patients with stable brain metastases at the time of inclusion derived relevant clinical benefit from T-DXd. This single-arm phase II study was developed to evaluate the direct activity of trastuzumab-deruxtecan on HER2-positive multiple BM.

2. STUDY DESIGN

This is an open label, non-comparative, single-centre, phase II study to evaluate the efficacy and safety of trastuzumab-deruxtecan (T-DXd; DS-8201a) in HER2-positive breast cancer patients with newly diagnosed or progressing brain metastases who are deemed candidates for systemic therapy. In this trial, trastuzumab-deruxtecan will be administered at a dose of 5.4 mg/kg bodyweight on day one of each cycle once every three weeks until progression, inacceptable toxicity or withdrawal (Figure 1). Before the first administration of the IMP, a cranial MRI and a CT scan of chest and abdomen will be conducted with further workup if indicated. Staging investigations will be repeated before the third and fifth treatment cycle and every nine weeks thereafter or whenever symptoms of disease progression occur.

2.1 STUDY OBJECTIVES

2.1.1. Primary Objective

To evaluate the ability of trastuzumab-deruxtecan to induce CNS responses in patients with HER2-positive breast cancer and newly diagnosed or progressive brain metastases.

2.1.2. Secondary Objectives

To evaluate the activity of trastuzumab-deruxtecan on extracranial disease, safety and tolerability of trastuzumab-deruxtecan in the patient population and QoL of study participants.

2.1.3. *Exploratory Objectives*

To evaluate biomarkers associated with response to trastuzumab-deruxtecan.

2.2. *STUDY ENDPOINTS*

2.2.1. *Primary Endpoints*

The primary endpoint of this study is the rate of best responses of BM at any assessment after the administration of at least one cycle of the IMP defined as CR, PR, SD and PD according to the RANO-BM criteria and as determined by the local investigator.

2.2.2. *Secondary Endpoints*

The secondary endpoints of this study consist of Clinical Benefit Rate CNS (CBR CNS as defined by RANO-BM; CR+PR+SD ≥6 months), extracranial response rate defines as CR, PR, SD and PD according to RECIST 1.1 criteria, progression-free survival defined as the interval from study inclusion until progression or death, time-to-WBRT defined as the interval from study inclusion until WBRT, overall survival defined as the interval from study inclusion until death, safety and QoL.

2.2.3. *Exploratory Endpoints*

Exploratory endpoints of this study include the number of *HER/neu* gene copies and the rate of TILs. For ancillary biomarker studies, blood samples (3 ml EDTA, 3 ml serum) will be drawn before administration of the IMP at cycles 1 and 4 and at EOT.

3. STUDY DURATION

- Duration of the study: 3 years
- Recruitment period: 30 months
- This study is expected to start in Q3 2020; last patient last visit (LPLV) is expected in Q3 2022.

4. NUMBER OF CENTRES

This is a single centre study conducted at the Medical University of Vienna/Vienna General Hospital, Department of Internal Medicine I, Division of Oncology.

5. SELECTION CRITERIA

5.1. TOTAL NUMBER OF PATIENTS

A maximum number of 15 evaluable patients will be enrolled based upon a Simon's optimal two-stage design; in stage 1, 6 patients will be accrued, with accrual of additional 9 patients up in stage 2 according to the number of responses seen in stage 1.

5.2. INCLUSION CRITERIA

To be eligible for inclusion, each patient must fulfil all of the following criteria:

- Histologically confirmed breast cancer
- Radiologically documented metastatic disease
- HER2-positive as defined by IHC 3+ and/or *HER2/neu* gene amplification
- Newly diagnosed brain metastases or brain metastases progressing after prior local therapy
- Measurable disease (RANO-BM criteria)
- No indication for immediate local treatment
- No indication of leptomeningeal disease
- KPS >70%, ECOG <2
- Indication for systemic anti-HER2 treatment
- Prior exposure to trastuzumab and pertuzumab
- Prior exposure to T-DM1 allowed

- Life expectancy of at least 3 months
- Age ≥18 years
- Patient must be able to tolerate therapy, and have adequate cardiac function (defined by baseline left ventricular ejection fraction ≥50%)
- Adequate bone-marrow, liver and kidney function
- Adequate treatment washout period before enrolment, defined as:
  - Major Surgery: ≥4 weeks
  - Radiation therapy: ≥4 weeks
  - Chemotherapy, small-molecule targeted agents, anticancer hormonal therapy: ≥3 weeks
  - Antibody-based treatment: ≥4 weeks
- Patient must be capable of understanding the purpose of the study and have given written informed consent

5.3. EXCLUSION CRITERIA

Patients who fulfil any of the following criteria will be excluded:

- Metastatic breast cancer other than HER2-positve disease
- Use of any investigational agent within 28 days prior to initiation of treatment
- History of malignancy other than squamous cell carcinoma, basal cell carcinoma of the skin or carcinoma in situ of the cervix within the last 3 years including contralateral breast cancer
- Major surgery, other than diagnostic surgery, within the last 4 weeks
- Indication for immediate local therapy by local standard
- Leptomeningeal involvement
- Other anticancer therapy, including cytotoxic, targeted agents, immunotherapy, antibody, retinoid, or anti-cancer hormonal treatment
- Concomitant radiotherapy
- Prior radiotherapy to the thorax other than breast irradiation or irradiation of bone metastases

- A history of uncontrolled seizures, central nervous system disorders or psychiatric disability judged by the investigator to be clinically significant and adversely affecting compliance to study drugs

- Clinically significant cardiac disease including unstable angina, acute myocardial infarction within six months prior to randomization, congestive heart failure (NYHA III-IV), left ventricular ejection fraction <50%, arrhythmia unless controlled by therapy, with the exception of extra systoles or minor conduction abnormalities, and long QT syndrome (QTc interval >450 ms)

- Subjects who have current active hepatic or biliary disease (with exception of patients with Gilbert's syndrome, asymptomatic gallstones, liver metastases or stable chronic liver disease per investigator assessment) including acute and chronic infections with hepatitis B and C

- Inadequate haematological status at baseline prior to study entry: Dependency on red blood cell and/or platelet transfusions, ANC (absolute neutrophil count (segmented + bands) <1.0 x 10^9/L; platelets <100 x 10^9/L

- Inadequate kidney function: serum-creatinine >1.5 times upper normal limit

- Hepatic dysfunction: total bilirubin >1.5 times upper normal limit (>3 in patients with liver metastases or known history of Gilbert's disease); ALT, AST >3 times upper normal limit (>5 in patients with liver metastases); serum albumin <2.5 g/dL; INR ≥1.5

- Clinically severe pulmonary compromise resulting from intercurrent pulmonary illnesses including, but not limited to, any underlying pulmonary disorder (i.e. pulmonary emboli within three months of the study enrolment, severe asthma, severe COPD, restrictive lung disease, pleural effusion etc.), and any autoimmune, connective tissue or inflammatory disorders with pulmonary involvement (i.e. rheumatoid arthritis, Sjogren's syndrome, sarcoidosis etc.), or prior pneumonectomy

- Subjects with bronchopulmonary disorders who require intermittent use of bronchodilators (such as albuterol) will not be excluded from this study

- Patients with active opportunistic infections

- Known HIV infection

- Concomitant treatment with chloroquine or hydroxychloroquine
- Pregnant or lactating women. Women with childbearing potential must have a negative pregnancy test at screening
- Women with childbearing potential, including women whose last menstrual period was less than one year prior to screening, unable or unwilling to use adequate contraception from study start to one year after the last dose of protocol therapy. Acceptable contraception methods included the application of an intrauterine device, barrier method or total abstinence
- Patients with known hypersensitivity to trastuzumab
- Patients not able to provide written informed consent
- Patients with known substance abuse or any other medical conditions such as clinically significant cardiac or psychological conditions, that may, in the opinion of the investigator, interfere with the subject's participation in the clinical study or evaluation of the clinical study results
- Patients requiring concomitant use of chronic systemic (IV or oral) corticosteroids at doses higher than 4 mg dexamethasone per day or other immunosuppressive medications except for managing adverse events; (inhaled steroids or intra articular steroid injections are permitted in this study)

6. DISEASE EVALUATION

6.1 PRIMARY ACTIVITY PARAMETERS

Response to treatment will be assessed for the first time within ten days before the third administration of trastuzumab-deruxtecan. At this assessment, a cranial MRI and a CT-scan of the chest and abdomen/pelvis will be performed with further workup if indicated. Any other instrumental investigations found to be abnormal at baseline evaluation prior to study entry will be repeated.

Patients with complete remission, partial response or stable disease will continue treatment per protocol, while patients progressing will be taken off study (at the assessment time points during the treatment period of the study).

2nd assessment: The second response assessment will be conducted within ten days before the fifth administration of trastuzumab-deruxtecan

Further assessment: Further response assessments will be conducted every nine weeks (e.g. before the eight, eleventh, fourteenth cycle)

In addition, reassessment should always be performed in case of symptoms indicating disease progression.

6.1.1 Response criteria (BM)

The rate of objective responses of BM will be assess using objective radiological criteria (Response Assessment in Neuro-Oncology; RANO) [23] according to the definitions of CR, PR, SD and PD.

Target lesions

- <u>Complete remission (CR):</u> Disappearance of all CNS target lesions sustained for at least 4 weeks; with no new lesions, no use of corticosteroids, and patient is stable or improved clinically.
- <u>Partial remission (PR):</u> At least a 30% decrease in the sum longest diameter of CNS target lesions, taking as reference the baseline sum longest diameter sustained for at least 4 weeks; no new lesions; stable to decreased corticosteroid dose; stable or improved clinically.
- <u>Stable disease (SD):</u> Neither sufficient shrinkage to qualify for partial response nor sufficient increase to qualify for progressive disease, taking as reference the smallest sum longest diameter while on study.
- <u>Progressive disease (PD):</u> At least a 20% increase in the sum longest diameter of CNS target lesions, taking as reference the smallest sum on study (this includes the baseline sum if that is the smallest on study). In addition to the relative increase of 20%, at least one lesion must increase by an absolute value of 5 mm or more to be considered progression.

Non-target lesions

- Complete remission (CR): Requires all of the following: disappearance of all enhancing CNS non-target lesions, no new CNS lesions.
- Non-CR/Non-PD: Persistence of one or more non-target CNS lesion or lesions.
- Progressive disease (PD): Any of the following: unequivocal progression of existing enhancing non-target CNS lesions, new lesion(s) (except while on immunotherapy-based treatment), or unequivocal progression of existing tumour-related non-enhancing (T2/FLAIR) CNS lesions. In the_case of immunotherapy-based treatment, new lesions alone may not constitute progressive disease.

6.1.2 Response criteria (extracranial disease)

The rate of objective extracranial responses will be assessed using objective radiological criteria (Response Evaluation Criteria In Solid Tumors; RECIST 1.1) [30] in patients with measurable disease according to the definitions of CR, PR, SD and PD.

Target lesions:

- Complete remission (CR): Disappearance of all target lesions. Any pathological lymph node (whether target or non-target) must have reduction in short axis to <10 mm.
- Partial remission (PR): At least a 30% decrease in the sum of diameter of target lesions, taking as reference the baseline sum diameters.
- Stable disease (SD): Neither sufficient shrinkage to qualify for PR nor sufficient increase to qualify for PD, taking as reference the smallest sum diameter while on study.
- Progressive disease (PD): At least 20% increase in the sum of diameters of target lesions, taking as reference the smallest sum on study (this includes the baseline sum if that is the smallest sum on study) In addition of to the relative increase of 20 percent, the sum must also demonstrate an absolute increase of at least 5 mm. The appearance of 1 or more new lesions is also considered progression.

Non-target lesions:

- Complete remission (CR): Disappearance of all non-target lesions and normalisation of tumour marker level. All lymph nodes must be non-pathological in size (< 10 mm short axis).

- Non-CR/Non-PD: Persistence of one or more non-target lesion(s) and/or maintenance of tumour marker level above normal limits.
- Progressive disease (PD): Unequivocal progression of existing non-target lesions. The appearance of one or more new lesions is also considered progression.

Patients progressing during treatment will be taken off study and treated at the judgement of the clinician. Patients progressing in the brain may continue on trastuzumab-deruxtecan after local therapy in the absence of extracranial disease progression.

7. STUDY MEDICATION

7.1 TRASTUZUMAB-DERUXTECAN

See also 1.2 "Trastuzumab-Deruxtecan Background and Rationale"

7.1.1 Formulation

Trastuzumab deruxtecan drug product is supplied in a single-use vial as 100 mg of DS-8201a in a sterile lyophilized powder dosage form (Lyo-DP) to be reconstituted with 5 mL of water for injection to 20 mg/mL, manufactured using DS2. Lyo-DP is a sterile lyophilized powder in an amber glass vial for intravenous infusion. Prior to use, Lyo-DP is reconstituted with 5 mL of water for injection to provide a solution with the concentration of 20 mg/mL DS-8201a in a buffer containing L-histidine, L-histidine hydrochloride monohydrate, sucrose, and polysorbate 80. Each vial contains 100 mg of DS-8201a.

7.1.2 Preparation

The instructions for the preparation of the trastuzumab-deruxtecan solution are found in the pharmacy instructions or in the IB.

7.1.3. Therapeutic regimen and dose

Trastuzumab-deruxtecan is administered at a dose of 5.4 mg/kg bodyweight as an intravenous infusion every 3 weeks (21-day cycle). First administration should occur a maximum of 7 days after enrolment.

The initial dose should be administered as a 90 minutes intravenous infusion. Patients should be observed during the infusion and for at least 90 minutes following the initial infusion for fever, chills, or other infusion-related reactions. The infusion site should be closely monitored for possible subcutaneous infiltration during administration. If the prior infusion was well tolerated, subsequent doses of T-DXd may be administered as 30 minutes infusions.

Patients should be observed during the infusion and for at least 30 minutes after infusion. The infusion rate of T-DXd should be slowed or interrupted if the patient develops infusion-related symptoms. T-DXd should be discontinued in case of life-threatening infusion reactions. Medicinal products to treat allergic/anaphylactic infusion reactions, as well as emergency equipment should be available for immediate use at site.

T-DXd is administered until disease progression (either in the brain or systemic), intolerable toxicity or subject withdrawal. Patients that progress in the brain but still have controlled systemic disease, and who receive local brain treatment, can continue to receive trastuzumab-deruxtecan off study until systemic progression after receiving local treatment.

7.1.4. Packaging & Labelling

The sponsor is responsible for providing the shipping orders. T-DXd will be provided by the manufacturer and labelled by the sponsor of this trial.

The IMP will be provided free of charge to the sponsor in individual pharmaceutical packings. Each pharmaceutical packing will identify the contents as study medication and bear protocol number. In addition, the label will bear the sponsors name, quantity contained, a package number and the standard caution statement as follows: Caution: New drug - Limited by Federal law to investigational use. The study drug label must be clearly visible.

7.1.5. Receipt of study drug

The hospital pharamcist is responsible for taking an inventory of each shipment of study drug received and for comparing it with the accompanying study drug accountability form. The pharmacist will verify the accuracy of the information on the form, sign, and date it, retain a copy in the study file. The numbers of pharmaceutical packings must be recorded when drug is received and dispensed.

7.1.6. Storage

Trastuzumab-deruxtecan will be stored at the hospital pharmacy in a secure area in accordance with Good Clinical Practice (GCP) and Good Manufacturing Practice (GMP) requirements and must be accessible to authorised personnel only. The storage condition for Lyo-DP is 2°C to 8°C (protected from light).

7.1.7. Unused study drug supplies

Unused study drug will be destroyed due to local SOPs. If any study drug is lost or damaged, its disposition should be documented in the source documents and Drug Dispensing Log.

8. SCREENING, ELIGIBILITY & TREATMENT

The Investigator is responsible for keeping a record of all subjects who sign an Informed Consent Form for entry into the study. All participants will be screened for eligibility. Investigations at baseline and during the study are outlined in Table 1. Study Assessments, unless otherwise specified, must take place within 28 days prior to initiation of therapy. Screening pregnancy tests for women of child-bearing potential must occur within three days before first application of IMP.

8.1 STUDY TREATMENT

In this trial, T-DXd will be administered at a dose of 5.4 mg/kg bodyweight once every three weeks by intravenous infusion until disease progression, inacceptable toxicity or withdrawal. A detailed summary of assessment schedules is provided in Table 1 and section 6.1.

8.2 STUDY PROCEDURES

Table 1 provides a detailed overview of study procedures at baseline (within 28 days of the first administration of the study drug) and the required investigations during the study. cMRI and CT of chest and abdomen required for study inclusion need not to be repeated if the interval from the investigation to the first administration of the study drug is ≤28 days. In women of childbearing potential, a pregnancy test not older than 72 hours is required before the first administration of the study drug.

8.3 DOSE CONTINUATION, MODIFICATION & INTERRUPTION

Subjects will be evaluated for AEs at each visit with the NCI CTCAE version 5.0 used as a guide for the grading of severity.

All dose modifications (interruption, reduction and/or discontinuation) should be based on the worst preceding toxicity (NCI CTCAE version 5.0). Specific criteria for interruption, re-initiation, dose reduction and/or discontinuation of trastuzumab deruxtecan are listed in table below, which is applicable only to TEAEs that are assessed as related to use of T-DXd by the investigator(s). For non-drug related TEAEs, standard clinical practice should be followed. In addition, appropriate clinical experts should be consulted as deemed necessary.

8.3.1. Dose Reduction Levels of Trastuzumab-deruxtecan

Starting Dose	***Dose Level -1***	***Dose Level -2***
5.4 mg/kg bodyweight	*4.4 mg/kg bodyweight*	*3.2 mg/kg bodyweight*

Once the dose of T-DXd has been reduced because of toxicity, all subsequent cycles should be administered at that lower dose level unless further dose reduction is required. More than 2 dose reductions are not allowed, and the subject will be withdrawn from the study treatment if further toxicity meeting the requirement for dose reduction occurs. T-DXd dose increases are not allowed in the study.

8.3.2. Dose Interruption and Modification /Toxicity Management Guidelines

A dose can be delayed for up to 28 days (Cycle Day 50) from the planned date of administration. If a subject is assessed as requiring a dose delay of longer than 28 days, the subject will be withdrawn from the study.

Treatment cycles for a subject for whom T-DXd dosing is temporarily withheld for any reason may have future cycles scheduled based on the date of the last T-DXd dose. All confirmed infections with SARS-CoV2 must be recorded in the study clinical database.

Worst toxicity CTCAE v5.0 Grade<br><br>***(unless otherwise specified)***	***Dose or schedule modification for DS-8201a***
No toxicity	*Maintain dose and schedule*
Infusion-Related Reaction	
Grade 1<br><br>*(Mild transient reaction; infusion interruption not indicated; intervention not indicated)*	*If infusion related reaction (such as fever and chills, with and without nausea/vomiting, pain, headache, dizziness, dyspnea, hypotension) is observed during administration, the infusion rate should be reduced by 50% and subjects should be closely monitored.*<br><br>*If no other reactions appear, the subsequent infusion rate could be resumed at the initial planned rate.*
Grade 2<br><br>*(Therapy or infusion interruption indicated but responds promptly to symptomatic treatment (e.g., antihistamines, nonsteroidal anti-inflammatory drugs (NSAIDs), narcotics, IV fluids); prophylactic medications indicated for ≤ 24 hrs)*	*Administration of DS-8201a should be interrupted and symptomatic treatment started (e.g. antihistamines, NSAIDs, narcotics, IV fluids).*<br><br>*If the event resolves or improves to grade 1, infusion can be re-started at a 50% reduced infusion rate.*<br><br>*Subsequent administrations should be conducted at the reduced rate.*
Grade 3 or 4<br><br>*(Prolonged or life-threatening consequences, urgent intervention indicated)*	*Administration of DS-8201a should be discontinued immediately and permanently.*<br><br>*Urgent intervention indicated. Antihistamines, steroids, epinephrine, bronchodilators, vasopressors, intravenous fluid therapy, oxygen inhalation etc., should be administered.*
Hematologic Toxicity	
Neutrophil Count Decrease and/or White Blood Cell Count Decrease	
Grade 3	*Delay dose until resolved to ≤ Grade 2, then maintain dose*
Grade 4	*Delay dose until resolved to ≤ Grade 2,*<br><br>*Reduce dose 1 level*
Febrile Neutropenia<br><br>*(absolute neutrophil count < 1 x 10^9/L, fever > 38.3°C or a sustained*	*Delay dose until resolved,*<br><br>*Reduce dose by 1 level*

Worst toxicity CTCAE v5.0 Grade<br><br>***(unless otherwise specified)***	***Dose or schedule modification for DS-8201a***
temperature of ≥ 38 °C for more than one hour)	
Lymphocyte Count Decreased	
Grade 1 to Grade 3<br><br>*Lymphopenia*	*No dose modification*
Grade 4<br><br>*(< 0.2 x 10^9/L)*	*Delay dose until resolved to ≤ Grade 2:*<br><br>- *If resolved in ≤ 14 days from day of onset, maintain dose*<br>- *If resolved in > 14 days from day of onset, reduce dose 1 level*
Anemia	
Grade 3<br><br>*(Hemoglobin (Hb) <8.0 g/dL); transfusion indicated*	*Delay dose until resolved to ≤ Grade 2, then maintain dose*
Grade 4<br><br>*Life threatening consequences; urgent intervention indicated*	*Delay dose until resolved to ≤ Grade 2, then reduce dose 1 level*
Platelet Count Decreased	
Grade 3<br><br>*(platelets <50 – 25 x 10^9/L)*	*Delay dose until resolved to ≤ Grade 1:*<br><br>- *If resolved in ≤ 7 days from day of onset, maintain dose*<br>- *If resolved in > 7 days from day of onset, reduce dose 1 level*
Grade 4<br><br>*(platelets < 25 x 10^9/L)*	*Delay dose until resolved to ≤ Grade 1, then reduce dose 1 level*
Cardiac Toxicity	
Symptomatic congestive heart failure (CHF)	*Discontinue subject from study treatment*

Worst toxicity CTCAE v5.0 Grade<br><br>***(unless otherwise specified)***	***Dose or schedule modification for DS-8201a***
Decrease in Left ventricle ejection fraction (LVEF) 10-20% (absolute value), but LVEF > 45%	*Continue treatment with DS-8201a*
LVEF 40% to ≤ 45% and decrease is < 10% (absolute value) from baseline	*Continue treatment with DS-8201a*<br><br>*Repeat LVEF assessment within 3 weeks*
LVEF 40% to ≤ 45% and decrease is ≥ 10% (absolute value) from baseline	*Interrupt DS-8201a dosing*<br><br>*Repeat LVEF assessment within 3 weeks.*<br><br>*If LVEF has not recovered to within 10% from baseline absolute value, discontinue subject from study treatment*<br><br>*If LVEF recovers to within 10% from baseline, resume study drug treatment*
LVEF < 40% or > 20% (absolute value) drop from baseline	*Interrupt trastuzumab deruxtecan dosing*<br><br>*Repeat LVEF assessment within 3 weeks.*<br><br>*If LVEF < 40% or > 20% drop from baseline is confirmed, discontinue subject from study treatment*
ECG QTc Prolongation	
Grade 3<br><br>*(Average QTc> 500 ms or >60 ms change from baseline)*	*Delay dose until resolved to ≤ Grade 1 (QTc ≤480 ms), determine if another medication the subject was taking may be responsible and can be adjusted or if there are any changes in serum electrolytes that can be corrected, then if attributed to trastuzumab deruxtecan, reduce dose 1 level*
Grade 4<br><br>*(Torsade de pointes or polymorphic ventricular tachycardia or signs/symptoms of serious arrhythmia)*	*Discontinue subject from study treatment*

Worst toxicity CTCAE v5.0 Grade<br><br>***(unless otherwise specified)***	***Dose or schedule modification for DS-8201a***
Pulmonary Toxicity	*If a subject develops radiographic changes potentially consistent with ILD/pneumonitis or develops an acute onset of new or worsening pulmonary or other related signs/symptoms such as dyspnea, cough or fever, rule out ILD/pneumonitis.*<br><br>*If the AE is confirmed to have an aetiology other than ILD/pneumonitis, follow the management guidance outlined in the "Other Non-Laboratory Adverse Events" dose modification section below.*<br><br>*If the AE is suspected to be ILD/pneumonitis, treatment with study drug should be interrupted pending further evaluations.*<br><br>*Evaluations should include:*<br><br>• *High resolution CT*<br>• *Pulmonologist consultation (Infectious Disease consultation as clinically indicated)*<br>• *Blood culture and CBC. Other BLOOD tests could be considered as needed*<br>• *Consider bronchoscopy and bronchoalveolar lavage if clinically indicated and feasible*<br>• *Pulmonary function tests and pulse oximetry (SpO2)*<br>• *Arterial blood gases if clinically indicated*<br>• *One blood sample collection for PK analysis as soon as ILD is suspected, if feasible.*<br><br>*Other tests could be considered, as needed.If the AE is confirmed to be ILD/pneumonitis, follow the management guidance as outlined below.*<br><br>*All events of ILD/pneumonitis regardless of severity or seriousness will be followed until resolution including after drug discontinuation.*
Grade 1	*The administration of trastuzumab-deruxtecan must be interrupted for any ILD/pneumonitis events regardless of grade.*

Worst toxicity CTCAE v5.0 Grade<br><br>***(unless otherwise specified)***	***Dose or schedule modification for DS-8201a***
	• *Monitor and closely follow-up in 2 to 7 days for onset of clinical symptoms and pulse oximetry*<br><br>• *Consider follow-up imaging in 1-2 weeks (or as clinically indicated).*<br><br>• *Consider starting systemic steroids (e.g. at least 0.5 mg/kg/day prednisone or equivalent) until improvement, followed by gradual taper over at least 4 weeks.*<br><br>• *If worsening of diagnostic observations despite initiation of corticosteroids, then follow Grade 2 guidelines.**<br><br>*For Grade 1 events, trastuzumab deruxtecan can be restarted only if the event is fully resolved to Grade 0:*<br><br>• *If resolved in ≤ 28 days from day of onset, maintain dose*<br>• *If resolved in > 28 days from day of onset, reduce dose 1 level*<br><br>*However, if the event grade 1 ILD/pneumonitis occurs beyond cycle day 22 and has not resolved within 49 days from the last infusion, the drug should be discontinued.*<br><br>** If participant is asymptomatic, then patient should still be considered as Grade 1 even if steroid treatment is given*
Grade 2	*Permanently discontinue subject from study treatment.*<br><br>*Promptly start and treat with systemic steroids (e.g., at least 1mg/kg/day prednisone or equivalent) for at least 14 days or*

Worst toxicity CTCAE v5.0 Grade<br><br>***(unless otherwise specified)***	***Dose or schedule modification for DS-8201a***
	until complete resolution of clinical and chest CT findings, then followed by a gradual taper over at least 4 weeks.<br><br>• *Monitor symptoms closely.*<br>• *Re-image as clinically indicated.*<br>• *If worsening or no improvement in clinical or diagnostic observations in 5 days*<br>• *Consider increasing dose of steroids (e.g., 2 mg/kg/day prednisone or equivalent) and administration may be switched to intravenous (e.g. methylprednisolone).*<br>• *Re-consider additional work-up for alternative aetiologies as described above.*<br>• *Escalate care as clinically indicated.*
Grade 3 and 4	*Permanently discontinue subject from study treatment.*<br><br>• *Hospitalization required.*<br>• *Promptly initiate empiric high-dose methylprednisolone IV treatment (e.g., 500-1000 mg/day for 3 days), followed by at least 1.0 mg/kg/day of prednisone (or equivalent) until clinical improvement, or at least 14 days or until complete resolution of clinical and chest CT findings, then followed by a* ***gradual taper*** *over at least 4 weeks.*<br>• *.*<br>• *Re-image as clinically indicated.*<br>• *If still no improvement within 3 to 5 days,*<br>• *Re-consider additional work-up for alternative etiologies as described above.*<br><br>*Consider other immuno-suppressants and/or treat per local practice.*
Ocular	

Worst toxicity CTCAE v5.0 Grade ***(unless otherwise specified)***	***Dose or schedule modification for DS-8201a***
Grade 3	*Delay dose until resolved to ≤ Grade 1:* *If resolved in ≤ 7 days from day of onset, maintain dose* *If resolved in > 7 days from day of onset, reduce dose 1 level*
Grade 4	*Discontinue subject from study treatment*
Blood creatinine increase	
Grade 3 (> 3.0 to 6.0 x upper limit of normal [ULN])	*Delay dose until resolved to ≤ Grade 2 or baseline, then reduce dose 1 level*
Grade 4 (> 6.0 x ULN)	*Discontinue subject from study treatment*
Hepatic Toxicity	
Aspartate aminotransferase (AST) or alanine aminotransferase (ALT) with simultaneous Total bilirubin increase (TBL)	
AST/ALT ≥ 3.0 x ULN with simultaneous TBL > 2.0 x ULN	*Delay study medication until drug-induced liver injury can be ruled out.* *If drug-induced liver injury is ruled out, the subject should be treated accordingly, and resumption of study drug may occur after discussion between the Investigator and Sponsor.* *If drug-induced liver injury cannot be ruled out from diagnostic workup, permanently discontinue study treatment.* *Monitor AST/ALT and TBL twice weekly until resolution or return to baseline.*
Aspartate aminotransferase (AST) or alanine aminotransferase (ALT)	
Grade 2 (>3.0 - 5.0 x ULN if baseline was normal; >3.0 - 5.0 x baseline if baseline was abnormal)	*No action for Grade 2 AST/ALT*
Grade 3 (>5.0 - 20.0 x ULN if baseline was normal; >5.0 - 20.0 x baseline if baseline was abnormal)	*Repeat testing within 3 days. Delay dose until resolved to ≤ Grade 1 if baseline ≤ 3 x ULN, otherwise delay dose until resolved to ≤ baseline, then:* *If resolved in ≤ 7 days from day of onset, maintain dose*

Worst toxicity CTCAE v5.0 Grade <br>***(unless otherwise specified)***	***Dose or schedule modification for DS-8201a***
In subjects without liver metastases and subjects with liver metastases and baseline level ≤ 3 x ULN	*If resolved in > 7 days from day of onset, reduce dose 1 level*
Grade 3: (>8.0 - 20.0 x ULN if baseline was normal; >8.0 - 20.0 x baseline if baseline was abnormal) <br>*In subjects with liver metastases, if the baseline level was > 3 x ULN*	*Repeat testing within 3 days. Delay dose until resolved to ≤ baseline level, then:* <br>*If resolved in ≤ 7 days from day of onset, maintain dose* *If resolved in > 7 days from day of onset, reduce dose 1 level*
Grade 4 (>20.0 x ULN if baseline was normal; >20.0 x baseline if baseline was abnormal)	*Discontinue subject from study treatment*
Blood bilirubin increase	
Grade 2 (>1.5 - 3.0 x ULN if baseline was normal; >1.5 - 3.0 x baseline if baseline was abnormal)	*If no documented Gilbert's syndrome or liver metastases at baseline, delay dose until resolved to ≤ Grade 1:* - *If resolved in ≤ 7 days from day of onset, maintain dose* - *If resolved in > 7 days from day of onset, reduce dose 1 level* <br>*If documented Gilbert's syndrome or liver metastases at baseline, continue study treatment*
Grade 3 (>3.0 - 10.0 x ULN if baseline was normal; >3.0 - 10.0 x baseline if baseline was abnormal)	*If no documented Gilbert's syndrome or liver metastases at baseline, repeat testing within 3 days. Delay dose until resolved to ≤ Grade 1:* - *If resolved in ≤ 7 days from day of onset, reduce dose 1 level* - *If resolved in > 7 days from day of onset, discontinue DS-8201a* <br>*If documented Gilbert's syndrome or liver metastases at baseline, repeat testing within 3 days. Delay dose until resolved to ≤ Grade 2:* - *If resolved in ≤ 7 days from day of onset, reduce dose 1 level* - *If resolved in > 7 days from day of onset, discontinue DS-8201a*
Grade 4 (>10.0 x ULN if baseline was normal; >10.0 x baseline if baseline was abnormal)	*Discontinue subject from study treatment*
Blood alkaline phosphatase Increase	

Worst toxicity CTCAE v5.0 Grade<br><br>***(unless otherwise specified)***	***Dose or schedule modification for DS-8201a***
Grade 3 (>5.0 - 20.0 x ULN if baseline was normal; >5.0 - 20.0 x baseline if baseline was abnormal)<br><br>*or*<br><br>*Grade 4 (>20.0 x ULN if baseline was normal; >20.0 x baseline if baseline was abnormal)*	*No modification unless determined by the Investigator to be clinically significant or life-threatening.*
Gastrointestinal	
Nausea	
Grade 3	*Delay dose until resolved to ≤ Grade 1*<br><br>*If resolved in ≤ 7 days from day of onset, maintain dose*<br>*If resolved in > 7 days from day of onset, reduce dose 1 level*
Diarrhoea/Colitis	
Grade 3	*Delay dose until resolved to ≤ Grade 1*<br><br>*If resolved in ≤ 3 days from day of onset, maintain dose*<br>*If resolved in > 3 days from day of onset, reduce dose 1 level*
Grade 4	*Discontinue subject from study treatment*
Other Laboratory Adverse Events	
Grade 3	*Delay dose until resolved to ≤ Grade 1 or baseline level:*<br><br>*If resolved in ≤ 7 days from day of onset, maintain dose*<br>*If resolved in > 7 days from day of onset, reduce dose 1 level*
Grade 4	*Discontinue subject from study treatment*
Other Non-Laboratory Adverse Events	
Grade 3	*Delay dose until resolved to ≤ Grade 1 or baseline:*<br><br>*If resolved in ≤ 7 days from day of onset, maintain dose*

Worst toxicity CTCAE v5.0 Grade<br><br>***(unless otherwise specified)***	***Dose or schedule modification for DS-8201a***
	If resolved in > 7 days from day of onset, reduce dose 1 level
Grade 4	*Discontinue subject from study treatment*

All dose modifications should be based on the worst preceding toxicity.
CTCAE: Common Terminology Criteria for Adverse Events.

In addition, Investigators may consider dose reductions or discontinuations of the study drug according to the subject's condition and after discussion with the Principal Investigator, Daiichi Sankyo's Medical Monitor or designee.

8.4 TREATMENT COMPLIANCE

At all times, study drug will be prepared and administered by study site personnel. Quantity and Lot-numbers of used vials/pharmaceutical packings must be recorded on “Drug Dispensing Logs” and in the source documents.

8.5 CONCOMITANT THERAPY

8.5.1 Recommended Concomitant Therapy

Subjects should receive full supportive care, including transfusions of blood and blood products when appropriate.

Hematopoietic growth factors may be used for prophylaxis or treatment based upon the investigator’s judgment. Prophylactic or supportive treatment of study-drug induced AEs (e.g. nausea) will be performed as per institutional guidelines. Concomitant use of dietary supplements, medications not prescribed by the investigator, and alternative/complementary treatments is discouraged, but not prohibited.

8.5.2 Prohibited Concomitant Therapy

- Concomitant use of other anti-cancer therapies, including radiation, chemotherapy or any other investigational agents is not permitted while subjects are receiving study drug during the treatment phase of the study.
- CYP3A4 strong inhibitors (include but are not limited to: boceprevir, clarithromycin, conivaptan, indinavir, itraconazole, ketoconazole, lopinavir/ritonavir, mibefradil, nefazodone, saquinavir, telaprevir, telithromycin, voriconazole). Consult with your local resources as needed to evaluate potential CYP3A4 inhibitors
- If concomitant use of strong CYP3A4 inhibitors is unavoidable, consider delaying DS-8201a treatment until the strong CYP3A4 inhibitors have cleared from the circulation (approximately 3 elimination half-lives of the inhibitors) when possible. If a strong CYP3A4 inhibitor is co-administered and DS-8201a treatment cannot be delayed, patients should be closely monitored for adverse reactions
- Organic anion transporting polypeptide (OATP) 1B inhibitors (include but are not limited to: atazanavir, clarithromycin, cyclosporine, erythromycin, gemfibrozil, lopinavir, rifampin, ritonavir, simeprevir). Consult with your local resources as needed to evaluate potential OATP1B inhibitors
- If concomitant use of OATP1B inhibitors is unavoidable, consider delaying DS-8201a treatment until the OATP1B inhibitors have cleared from the circulation (approximately 3 x the elimination half-life of the inhibitors) as far as possible

- If an OATP1B inhibitor is co-administered and DS-8201a treatment cannot be delayed, patients should be closely monitored for adverse reactions
- Concomitant use of foods or beverages containing grapefruit

9. PREMATURE WITHDRAWAL

Any patients who experience a serious adverse event may be withdrawn at any time from the study at the discretion of the investigator. If trastuzumab-deruxtecan is stopped for any reason other than progression (either toxicity or voluntary withdrawal from study treatment) patients will have the planned safety follow-up visit and enter in efficacy follow-up with cranial MRI and CT scans of chest and abdomen as well as other investigations according to the same timetable as if the treatment interruption had not occurred. This follow-up is to continue until disease progression or until voluntary withdrawal of the patient from the study. In case the follow-up is impossible (due to patient refusing to perform assessments or other reasons), survival data can be collected at the defined timepoints via chart review or telephone.

9.1 PREMATURE TRIAL TERMINATION

If any serious disadvantage of the treatment becomes evident during the clinical trial, therapy will be terminated. In this case the necessary procedures will be arranged to ensure protection of the subjects' interests.

9.2 REPLACEMENT POLICY

A total of 15 patients will be enrolled into the study; 6 evaluable patients (stage 1), with accrual of additional 9 patients (stage 2) according to the number of responses seen in stage 1. If patients are withdrawn within the first 9 weeks of the study (i.e. before the first response evaluation) for reasons other than progression or death, they will be replaced.

10. STATISTICAL CONSIDERATIONS

10.1 SAMPLE SIZE CALCULATION

This is a phase II study for proof of principle on the activity of trasuzumab-deruxtecan to induce objective responses in patients with HER2-positive metastatic breast cancer with newly diagnosed or progressing brain metastases based upon a Simon's two-stage design [31].

A response rate of 61% would be considered as indicating clinically relevant activity in this patient population. A response rate of <26%, on the other hand, must be considered clinically unmeaningful (null hypothesis). These assumptions are based on previous studies conducted in HER2-positive breast cancer patients with brain metastases. In the LANDSCAPE trial evaluating lapatinib plus capecitabine as upfront systemic therapy in patients with newly diagnosed BM, a response rate of 66% was observed; in contrast, the same regimen yielded a response rate of 20% in progressive BM after prior local therapy [32]. In a mixed population of patients with newly-diagnosed or progressive BM treated with T-DM1, a response rate of 30% was reported [22]; this study, however, included only two patients with newly diagnosed BM. Therefore, any response rate below 26% was considered to indicate little benefit of T-DXd over the currently available drugs in this specific population. In the phase II DESTINY-Breast01 study [28], T-DXd yielded an extracranial response rate of 60.9% in an extremely heavily pretreated population of HER2-positive MBC patients with virtually all patients showing some degree of tumour shrinkage. As the participants of TUXEDO-1 are expected to be similarly heavily pretreated, a response rate >60% is believed to indicate relevant clinical activity of T-DXd also in brain metastases of HER2-positive breast cancer.

In the first stage, 6 patients will initially be accrued. If there are two or fewer responses in these 6 patients, the study will be stopped. Otherwise, 9 additional patients will be accrued for a total number of 15 patients. The null hypothesis will be rejected if 7 or more responses are observed in these 15 patients. This design yields a type I error rate of 5% and a power of 80% to reject the null-hypothesis.

P0=0.250, P1=0.610, Alpha=0.050, Beta=0.200

**N1**	**R1**	**N**	**R**	**Alpha**	**Beta**
6	2	15	6	0.050	0.200

N1 is the sample size in the first stage.

R1 is the drug rejection number in the first stage.

N is the combined sample size of both stages.

R is the combined drug rejection number after both stages.

Alpha is the probability of rejecting that P<=P0 when this is true.

Beta is the probability of rejecting that P>=P1 when this is true.

P0 is the response proportion of a poor drug.

P1 is the response proportion of a good drug.

Tolerability and Safety:

Endpoints are safety & tolerance of treatment in terms of haematologic and non-haematologic side effects as assessed by the investigators. Adverse events will also be summarised using frequency counts and percentages.

All patients who are eligible for the study and receive at least one dose of study drug l be will be included in the safety analysis.

Patients who drop out or die prior to the first response assessment will be included in the denominator when calculating the response rate. Any patients who enrol into the study but who receive no study medication will be excluded, but a sensitivity analysis will be conducted which will include all enrolled patients.An analysis of all patients undergoing treatment per protocol excluding drop out will also be performed

10.2 EFFICACY

10.2.1. Primary Endpoint

Rate of objective responses of BM as judged by best response during therapy i.e. complete remission (CR), partial remission (PR), stable disease (SD)/no change and progressive disease as defined by RANO criteria.

Response categories according to RANO-BM criteria will be summarised using frequency counts and percentages. Exact 95% two-sided confidence intervals will also be provided for the response rate.

10.2.2. Secondary Endpoints

Rate of objective responses of extracranial metastases as judged by best response during therapy i.e. complete remission (CR), partial remission (PR), stable disease (SD)/no change and progressive disease as defined by RECIST 1.1 criteria.

Response categories according to RECIST 1.1 criteria will be summarised using frequency counts and percentages. Exact 95% two-sided confidence intervals will also be provided for the response rate.

Progression-free survival and overall survival will be assessed using the Kaplan-Maier product-limit method. Differences in PFS and OS curves in subgroups will be analysed by the log-rank test. A competing-risk model including death or study withdrawal will be calculated to assess time-to-WBRT and differences in the respective curves will be analysed by the Gray’s test.

10.3 TOLERABILITY & SAFETY

10.3.1. Endpoint

Safety and tolerance of treatment in terms of haematologic and non-haematologic side effects as assessed by the investigators. Adverse events will also be summarised using frequency counts and percentages. MedDRA terminology will be used and grading of AEs will be performed according to NCI CTCAE v5.0.

10.4 QUALITY-OF-LIFE

10.4.1. Endpoint

Quality-of-Life (QoL) in patients receiving trastuzumab-deruxtecan for HER2-positive breast cancer brain metastases will be assessed with the EORTC QLQ-c30 questionnaire, the brain specific tool (BN20), and the breast specific tool (BR45) at baseline, before cycle 3 and 5 and every 9 weeks thereafter and at EOT.

10.5 EXPLORATORY ENDPOINTS

10.5.1. Endpoint

Analysis of biomarkers potentially associated with response to trastuzumab-deruxtecan.

10.6 ANALYSIS PLAN

All patients who are eligible for the study and receive at least one dose of study drug will be included in the analysis. Any patients who enrol into the study but who receive no study medication will be excluded, but a sensitivity analysis will be conducted which will include all enrolled patients. An analysis of all patients undergoing treatment per protocol excluding drop out will also be performed**.**

10.7 RELEVANT PROTOCOL DEVIATIONS

Major deviations regarding to patients' safety will lead to subject withdrawal.

All protocol deviations will be listed in the clinical study report (CSR).

10.8 MISSING, UNUSED AND SPURIOUS DATA

Missing values will not be imputed.

11. SAFETY

11.1 ADVERSE EVENTS

An adverse event (AE) is any noxious, unintended, or untoward medical occurrence occurring at any dose that may appear or worsen in a subject during a study. It may be a new intercurrent illness, a worsening concomitant illness, an injury, or any concomitant impairment of the subject's health, including laboratory test values (as specified by the criteria below), regardless of etiology. Any medical condition that was present prior to study treatment and that remains unchanged or improved should not be recorded as an AE. If there is a worsening of that medical condition this should be considered an AE. A diagnosis or syndrome should be recorded on the AE page of the Case Report Form rather than the individual signs or symptoms of the diagnosis or syndrome. All AEs will be recorded by the investigator from the time of signing the informed consent through to the end of the designated follow-up period.

A Treatment-emergent adverse event (TEAE) is defined as an AE that occurs, having been absent before the first dose of study drug, or has worsened in severity or seriousness after the initiating the study drug until 47 days after last dose of the study drug. SAEs with an onset or worsening 48 days or more after the last dose of study drug, if considered related to the study treatment, are also TEAEs.

11.2 SERIOUS ADVERSE EVENTS

A serious adverse event (SAE) is any AE which:

- Results in death
- Is life-threatening (i.e., in the opinion of the Investigator(s) the subject is at immediate risk of death from the AE)
- Requires inpatient hospitalization or prolongation of existing hospitalization
- Results in persistent or significant disability/incapacity (a substantial disruption of the subject's ability to conduct normal life functions)
- Is a congenital anomaly/birth defect
- Constitutes an important medical event

Important medical events are defined as those occurrences that may not be immediately life threatening or result in death, hospitalization, or disability, but may jeopardize the subject or require medical or surgical intervention to prevent one of the other outcomes listed above. Medical and scientific judgment should be exercised in deciding whether such an AE should be considered serious.

Events not considered to be SAEs are hospitalizations which: were planned before entry into the clinical study; are for elective treatment of a condition unrelated to the studied indication or its treatment; occur on an emergency outpatient basis and do not result in admission (unless fulfilling other criteria above); are part of the normal treatment or monitoring of the studied indication and are not associated with any deterioration in condition.

If an AE is considered serious, both the AE pages of the CRF and the SAE Report Form must be completed.

For each SAE, the Investigator will provide information on severity, start and stop dates, relationship to study drug, action taken regarding study drug, and outcome.

11.3 ADVERSE EVENT OF SPECIAL INTEREST

For the DS-8201a clinical program, based on the available pre-clinical data, review of the cumulative literature, reported toxicities for the same class of agents and biological plausibility, the following events are considered to be adverse events of special interest (AESI): Interstitial lung disease/pneumonitis, and LVEF decrease.

11.3.1. Interstitial Lung Disease/Pneumonitis

Clinical Summary:

Interstitial lung disease/pneumonitis is considered an important identified risk based on a comprehensive cumulative review of the available safety data from the clinical development program. Refer to the current IB for a summary of preliminary clinical study data.

Management Guidance:

ILD should be ruled out if a subject develops radiographic changes potentially consistent with ILD or develops an acute onset of new or worsening pulmonary or other related signs/symptoms such as dyspnoea, cough or fever. If the AE is confirmed to have an aetiology other than ILD, follow the management guidance outlined in the designated “Other Non-Laboratory Adverse Events” dose modification section of the study protocol.

If the AE is suspected to be ILD, treatment with study drug should be interrupted pending further diagnostic evaluations. Evalations, should include high resolution CT, pulmonologist consultation (infectious disease consultation as clinically indicated), blood culture and CBC (other blood tests could

be considered as needed), bronchoscopy and bronchoalveolar lavage if clinically indicated and feasible should be considered, pulmonary function tests and pulse oximetry (SpO_2), arterial blood gases if clinically indicated, One blood sample collection for PK analysis as soon as ILD is suspected, if feasible.

If the AE is confirmed to be ILD, follow the management guidance outlined in the designated "Pulmonary Toxicity" dose modification section of the study protocol. All events of ILD regardless of severity or seriousness will be followed until resolution including after drug discontinuation.

11.3.2. LVEF decrease

Clinical Summary:

LVEF decrease in association with DS-8201a treatment is considered to be an important potential risk based on the available pre-clinical data, literature and available safety information for drugs of a similar class. Refer to the current IB for a summary of preliminary clinical trial data.

Management Guidance:

LVEF will be measured by either ECHO or MUGA scan. All ECHOs/MUGAs will be evaluated by the Investigator or delegated physician for monitoring cardiac function.

Troponin will be measured at screening and EOT, and as needed based on subject reported cardiac signs or symptoms suggesting congestive heart failure, myocardial infarction, or other causes of cardiac myocyte necrosis.

Triplicate ECGs will be performed and standard ECG parameters will be measured, including RR, PR, QT intervals, and QRS duration. All ECGs must be evaluated by Investigator or delegated physician for the presence of abnormalities. Whether or not measurement is performed, date performed, results, and findings for each parameter is to be recorded in the CRF.

11.4 CLASSIFICATION OF SEVERITY

For both AEs and SAEs, the investigator must assess the severity of the event. The severity of adverse events (AEs) will be graded on a scale of 1 to 5 according to the National Cancer Institute (NCI) Common Terminology Criteria for Adverse Events Version 5.0 (NCI CTCAE). The NCI CTCAE V5.0 can be viewed

on-line at the following NCI: web site:
https://ctep.cancer.gov/protocolDevelopment/electronic_applications/ctc.htm#ctc_50.

If a specific event is not included in the NCI CTCAE toxicity scale, the following scale should be used to grade the event

Grade	Definition
1	**Mild** Awareness of sign, symptom, or event, usually transient, requiring no special treatment and generally not interfering with usual daily activities
2	**Moderate** Discomfort that causes interference with usual activities; usually ameliorated by basic therapeutic manoeuvres
3	**Severe** Incapacitating with inability to do usual activities or significantly affects clinical status and warrants intervention. Hospitalization may or may not be required
4	**Life-threatening** Immediate risk of death; requires hospitalization and clinical intervention
5	**Death**

11.5 CLASSIFICATION OF RELATIONSHIP/CAUSALITY OF ADVERSE EVENTS (AE/SAE) TO IMP

The Investigator must determine the relationship between the administration of study drug and the occurrence of an AE/SAE as Not Suspected or Suspected as defined below:

Not suspected:

The temporal relationship of the adverse event to study drug administration makes a causal relationship unlikely or remote, or other medications, therapeutic interventions, or underlying conditions provide a sufficient explanation for the observed event.

Suspected:

The temporal relationship of the adverse event to study drug administration makes a causal relationship possible, and other medications, therapeutic interventions, or underlying conditions do not provide a sufficient explanation for the observed event.

11.6 SAE AND SUSAR REPORTING

11.6.1. Reporting to the Regulatory Authority and the Ethics Committee

The sponsor will inform relevant Regulatory Authority and the Ethics Committee:

- Of all relevant information about suspected unexpected serious adverse reactions related to the study medication (SUSARs) that are fatal or life threatening as soon as possible, and in any case no later than seven days after knowledge of such a case. Relevant follow-up information for these cases will subsequently be submitted within an additional eight days.
- Of all other SUSARs as soon as possible, but within a maximum of fifteen days of first knowledge by the investigator.

11.6.2. Reporting to Daiichi Sankyo

The investigator will inform Daiichi Sankyo (email address: cspv-clinical@dsi.com) within 24 hours of the following types of events:

- All SAEs
- All potential ILD/pneumonitis cases should be reported within 24 hours including both serious and non-serious potential ILD/pneumonitis cases (potential ILD/pneumonitis is defined by the Event Adjudication Site Manual List of PTs).
- Pregnancies
- Hepatic events (both serious and non-serious) which meet the potential Hy's Law criteria defined as an elevated (ALT or AST) ≥ 3 x ULN and an elevated TBL > 2 x ULN that may occur either at different time points or simultaneously during the study. Overdose, defined as the accidental or intentional administration of any dose of a product that is considered both excessive and medically important. An "excessive and medically important" overdose includes any overdose in which either a serious adverse event, a non-serious adverse event, or no adverse event occurs and is considered by the Investigator as clinically relevant, i.e. poses an actual or potential risk to the subject.
- Overdose is always serious. By definition, an overdose is medically important, which meets the seriousness criterion of important medical event. An overdose can occur with or without an AE. AEs can either be serious or non-serious. Details of the overdose including DS-8201a dosage, clinical course, associated AEs, and outcome must be captured in the Narrative form of the CRF.

11.6.3. Immediate Reporting

The investigator will inform the pharmacovigilance officer for this study of all SAEs within 24 hours after knowledge of such a case in order that the sponsor can fulfil his regulatory reporting obligations within the required timeframes.

The IB will be provided by Daiichi-Sankyo in the currently available form.

11.7 PREGNANCIES

Female of Childbearing Potential:

Pregnancies and suspected pregnancies (including a positive pregnancy test regardless of age or disease state) of a female subject occurring while the subject is on study drug, or within 28 days of the subject's last dose of study drug, are considered events to be reported immediately to Sponsor. If the subject is on study drug, the study drug is to be discontinued immediately and the subject instructed to return any unused portion of the study drug to the Investigator. The pregnancy, suspected pregnancy, or positive pregnancy test must be reported to Sponsor.

The female should be referred to an obstetrician/gynecologist experienced in reproductive toxicity for further evaluation and counseling.

The investigator will follow the female subject until completion of the pregnancy, and must notify the Sponsor of the outcome of the pregnancy within 5 days or as specified below. The Investigator will provide this information as a follow-up to the initial pregnancy report.

If the outcome of the pregnancy meets the criteria for immediate classification as a SAE (i.e., spontaneous or therapeutic abortion [any congenital anomaly detected in an aborted fetus is to be documented], stillbirth, neonatal death, or congenital anomaly [including that in an aborted fetus]), the Investigator should follow the procedures for reporting SAEs.

All neonatal deaths that occur within 28 days of birth should be reported, without regard to causality, as SAEs. In addition, any infant death after 28 days that the Investigator suspects is related to the in utero exposure to the study drug should also be reported.

12. QUALITY CONTROL & QUALITY ASSURANCE

12.1 PERIODIC MONITORING

The designated monitor will contact and visit the investigator regularly and will be allowed to have access to all source documents needed to verify the entries in the CRFs and other protocol-related documents provided that subject confidentiality is maintained in agreement with local regulations. It will be the monitor's responsibility to inspect the CRFs at regular intervals throughout the study, to verify the adherence to the protocol and the completeness, consistency and accuracy of the data being entered on them. The monitoring standards require full verification for the presence of informed consent, adherence to the inclusion/exclusion criteria, documentation of SAEs and the recording of the main efficacy & safety endpoints.

To be GCP compliant at least 3 monitoring visits are scheduled. An initiation visit, one routine visit and a final visit after the last patient has finished the study. The monitor will provide a GCP-compliant monitoring report after each visit for the sponsor. The investigator will cooperate with the CRA to ensure that any identified discrepancies are resolved.

12.2 AUDITS & INSPECTIONS

The main purpose of an audit or inspection is to confirm that the rights and welfare of the subjects have been adequately protected, and that all data relevant for assessment of safety and efficacy of the investigational product have appropriately been reported.

The sponsor/investigator will permit trial related monitoring, audit, IRB/IEC review & regulatory inspections and provide direct access to study related-source data and source documents.

12.3 PUBLICATION OF STUDY RESULTS

The findings of this study will be published by the Principal Investigator in a scientific journal and presented at scientific meetings.

13. ETHICAL & LEGAL ASPECT

13.1 INFORMED CONSENT OF SUBJECTS

Following comprehensive instruction regarding the nature, significance, impact and risks of this clinical trial, the patient must give written consent to participation in the study.

- The patients are made aware of the fact that they can with-draw their consent – without giving reasons – at any time without their further medical care being influenced in any way

and they also receive a written patient information sheet in comprehensible language, explaining the nature and purpose of the study and its progress.

- The patients also must agree to the possibility of study-related data being passed on to relevant authorities.
- The patients will be informed in detail of their obligations in relation to the insurance in order not to jeopardize insurance cover.

13.2 ACKNOWLEDGEMENT/APPROVAL OF THE STUDY

The investigator submits this protocol and any related document provided to the subject (such as subject information used to obtain informed consent) to an Ethics Committee (EC) or Institutional Review Board (IRB). Approval from the committee must be obtained before starting the study, and should be documented in a dated letter to the investigator.

Adverse events - whether serious and/or unexpected, and possibly endangering the safety of the study participants - are likewise to be reported to the ethics committee.

The clinical trial will be performed in full compliance with the valid legal regulations according to the Drug Law (AMG - Arzneimittelgesetz) of the Republic of Austria.

The study will be notified to the Austrian Agency for Health and Food Safety (AGES) and registered to the European Clinical Trial Database (EudraCT) using the required forms.

13.3 PROTOCOL AMENDMENTS

Proposed amendments will be submitted to the appropriate CA and ECs. Substantial amendments may be implemented only after CA/EC approval has been obtained. Amendments that are intended to eliminate an apparent immediate hazard to subjects will be implemented prior to receiving CA/EC approval. However, in this case, approval must be obtained as soon as possible after implementation.

13.4 STUDY TERMINATION

If the investigator/sponsor decides to terminate the study before it is completed, he will ensure that adequate consideration is given to the protection of the subject interests. The investigator/ sponsor will notify the relevant CA and EC. Documentation will be filed in the TMF and the ISF.

13.5 CLINICAL STUDY REPORT (CSR)

Within one year after the final completion of the study, a full CSR will be written by the investigator or designee and submitted to the EC and the competent authority. The Principal Investigator will review and sign the final study report.

13.6 FINANCE & INSURANCE

13.6.1 Finance

Investigator Initiated trial; study drug is made available by the aponsor.

13.6.2 Insurance

During their participation in the clinical trial the patients will be insured as defined by legal requirements. The sponsor is providing insurance in order to indemnify (legal and financial coverage) the investigator/centre against claims arising from the study, except for claims that arise from malpractice and/or negligence. The compensation of the subject in the event of study-related injuries will comply with the applicable regulations.

Details on the existing patients' insurance:

Zürich Versicherungs-AG, Schwarzenbergplatz 15, 1010 Vienna; Policy: 07229622-2

These details are also provided in the patient information sheet.

13.7 ETHICS AND GOOD CLINICAL PRACTICE (GCP)

The investigator ensures that this study is conducted in full conformance with the principles of the current version from "Declaration of Helsinki", the ICH GCP Guidelines and the laws and regulations of Austria.

14. APPENDICES

14.1 ***APPENDIX I***

ECOG Performance Status Scale [33]

ECOG Scale	Performance Status
0	Fully active, able to carry out all pre-disease performance without restriction.
1	Restricted in physically strenuous activity, but ambulatory and able to carry out work of a light or sedentary nature, e.g. light housework, office work.
2	Ambulatory and capable of all selfcare, but unable to carry out any work activities. Up and about more than 50% of waking hours.
3	Capable of only limited selfcare, confined to bed or chair more than 50% of waking hours.
4	Completed disabled. Cannot carry out any selfcare. Totally confined to bed or chair.
5	Dead

ECOG and Karnofsky Performance Status:

ECOG 0 corresponds to Karnofsky Performance Status of	100-90
ECOG 1 corresponds to Karnofsky Performance Status of	80-70
ECOG 2 corresponds to Karnofsky Performance Status of	60-50
ECOG 3 corresponds to Karnfosky Performance Status of	40-30
ECOG 4 corresponds to Karnofsky Performance Status of	20-10
ECOG 5 corresponds to Karnofsky Performance Status of	0

14.2 APPENDIX II

Laboratory Parameter

Haematology:

Haemoglobin, Leucocytes, Neutrophiles abs., Lymphocytes abs., Monocytes abs., Eosinophiles abs., Basophiles abs., Platelets

Biochemistry:

Total protein, Albumin, CRP, IgG, IgA, IgM, ß2-Microglobulin, Glucose, Serum Creatinine, BUN, Uric acid, total bilirubin, ASAT (SGOT), ALAT (SGPT), γGT, Alkaline phosphatase, LDH, Cholesterol, Triglycerides, Sodium, Phosphat, Potassium, Calcium, Magnesium, Normotest, Fibrinogen.

13.3. APPENDIX III

DECLARATION OF HELSINKI

Amended at the 64th WMA General Assembly, Fortaleza, Brazil, October 2013 [34].

Preamble

1. The World Medical Association (WMA) has developed the Declaration of Helsinki as a statement of ethical principles for medical research involving human subjects, including research on identifiable human material and data.

The Declaration is intended to be read as a whole and each of its constituent paragraphs should be applied with consideration of all other relevant paragraphs.

2. Consistent with the mandate of the WMA, the Declaration is addressed primarily to physicians. The WMA encourages others who are involved in medical research involving human subjects to adopt these principles.

General Principles

3. The Declaration of Geneva of the WMA binds the physician with the words, “The health of my patient will be my first consideration,” and the International Code of Medical Ethics declares that, “A physician shall act in the patient’s best interest when providing medical care.”

4. It is the duty of the physician to promote and safeguard the health, well-being and rights of patients, including those who are involved in medical research. The physician’s knowledge and conscience are dedicated to the fulfilment of this duty.

5. Medical progress is based on research that ultimately must include studies involving human subjects.

6. The primary purpose of medical research involving human subjects is to understand the causes,

development and effects of diseases and improve preventive, diagnostic and therapeutic interventions (methods, procedures and treatments). Even the best proven interventions must be evaluated continually through research for their safety, effectiveness, efficiency, accessibility and quality.

7. Medical research is subject to ethical standards that promote and ensure respect for all human subjects and protect their health and rights.

8. While the primary purpose of medical research is to generate new knowledge, this goal can never take precedence over the rights and interests of individual research subjects.

9. It is the duty of physicians who are involved in medical research to protect the life, health, dignity,

integrity, right to self-determination, privacy, and con dentiality of personal information of research subjects. The responsibility for the protection of research subjects must always rest with the physician or other health care professionals and never with the research subjects, even though they have given consent.

10. Physicians must consider the ethical, legal and regulatory norms and standards for research involving human subjects in their own countries as well as applicable international norms and standards. No national or international ethical, legal or regulatory requirement should reduce or eliminate any of the protections for research subjects set forth in this Declaration.

11. Medical research should be conducted in a manner that minimises possible harm to the environment.

12. Medical research involving human subjects must be conducted only by individuals with the appropriate ethics and scientific education, training and qualifications. Research on patients or healthy volunteers requires the supervision of a competent and appropriately qualified physician or other health care professional.

13. Groups that are underrepresented in medical research should be provided appropriate access to

participation in research.

14. Physicians who combine medical research with medical care should involve their patients in research only to the extent that this is justified by its potential preventive, diagnostic or therapeutic value and if the physician has good reason to believe that participation in the research study will not adversely affect the health of the patients who serve as research subjects.

15. Appropriate compensation and treatment for subjects who are harmed as a result of participating in research must be ensured.

Risks, Burdens and Benefits

16. In medical practice and in medical research, most interventions involve risks and burdens.

Medical research involving human subjects may only be conducted if the importance of the objective outweighs the risks and burdens to the research subjects.

17. All medical research involving human subjects must be preceded by careful assessment of predictable risks and burdens to the individuals and groups involved in the research in comparison with foreseeable benefits to them and to other individuals or groups affected by the condition under investigation. Measures to minimise the risks must be implemented. The risks must be continuously monitored, assessed and documented by the researcher.

18. Physicians may not be involved in a research study involving human subjects unless they are confident that the risks have been adequately assessed and can be satisfactorily managed. When the risks are found to outweigh the potential bene ts or when there is conclusive proof of definitive

outcomes, physicians must assess whether to continue, modify or immediately stop the study.

Vulnerable Groups and Individuals

19. Some groups and individuals are particularly vulnerable and may have an increased likelihood of being wronged or of incurring additional harm. All vulnerable groups and individuals should receive specifically considered protection.

20. Medical research with a vulnerable group is only justified if the research is responsive to the health needs or priorities of this group and the research cannot be carried out in a non-vulnerable group. In addition, this group should stand to bene t from the knowledge, practices or interventions that result from the research.

Scientific Requirements and Research Protocols

21. Medical research involving human subjects must conform to generally accepted scientific principles, be based on a thorough knowledge of the scientific literature, other relevant sources of information, and adequate laboratory and, as appropriate, animal experimentation. The welfare of animals used for research must be respected.

22. The design and performance of each research study involving human subjects must be clearly described and justified in a research protocol. The protocol should contain a statement of the ethical considerations involved

and should indicate how the principles in this Declaration have been addressed. The protocol should include information regarding funding, sponsors, institutional affiliations, potential conflicts of interest, incentives for subjects and information regarding provisions for treating and/or compensating subjects who are harmed as a consequence of

participation in the research study. In clinical trials, the protocol must also describe appropriate arrangements for post-trial provisions.

Research Ethics Committees

23. The research protocol must be submitted for consideration, comment, guidance and approval to the concerned research ethics committee before the study begins. This committee must be transparent in its functioning, must be independent of the researcher, the sponsor and any other undue influence and must be duly qualified. It must take into consideration the laws and regulations of the country or countries in which the research is to be performed as well as applicable international norms and standards but these must not be allowed to reduce or eliminate any of the protections for research subjects set forth in this Declaration. The committee must have the right to monitor ongoing studies. The researcher must provide monitoring information to the committee, especially information about any serious adverse events. No amendment to the

protocol may be made without consideration and approval by the committee. After the end of the study, the researchers must submit a nal report to the committee containing a summary of the study's findings and conclusions.

Privacy and Confidentiality

24. Every precaution must be taken to protect the privacy of research subjects and the confidentiality of their personal information.

Informed Consent

25. Participation by individuals capable of giving informed consent as subjects in medical research must be voluntary. Although it may be appropriate to consult family members or community leaders, no individual capable of giving informed consent may be enrolled in a research study unless he or she freely agrees.

26. In medical research involving human subjects capable of giving informed consent, each potential subject must be adequately informed of the aims, methods, sources of funding, any possible conflicts of interest, institutional affiliations of the researcher, the anticipated benefits and potential risks of the study and the discomfort it may entail, post-study provisions and any other relevant aspects of the study. The potential subject must be informed of the right to refuse to participate in the study or to withdraw consent to participate at any time without reprisal. Special attention should be given to the specific information needs of individual potential subjects as well as to the methods used to deliver the information.

After ensuring that the potential subject has understood the information, the physician or another

appropriately qualified individual must then seek the potential subject's freely-given informed consent, preferably in writing. If the consent cannot be expressed in writing, the non-written consent must be formally documented and witnessed.

All medical research subjects should be given the option of being informed about the general outcome and results of the study.

27. When seeking informed consent for participation in a research study the physician must be particularly cautious if the potential subject is in a dependent relationship with the physician or may consent under duress. In such situations the informed consent must be sought by an appropriately qualified individual who is completely independent of this relationship.

28. For a potential research subject who is incapable of giving informed consent, the physician must seek informed consent from the legally authorised representative. These individuals must not be included in a research study that has no likelihood of bene t for them unless it is intended to promote the health of the group represented by the potential subject, the research cannot instead be performed with persons capable of providing informed consent, and the research entails only minimal risk and minimal burden.

29. When a potential research subject who is deemed incapable of giving informed consent is able to give assent to decisions about participation in research, the physician must seek that assent in addition to the consent of the legally authorised representative. The potential subject's dissent should be respected.

30. Research involving subjects who are physically or mentally incapable of giving consent, for example, unconscious patients, may be done only if the physical or mental condition that prevents giving informed consent is a necessary characteristic of the research group. In such circumstances the physician must seek informed consent from the legally authorised representative. If no such representative is available and if the research cannot be delayed, the study may proceed without informed consent provided that the specific reasons for involving subjects with a condition that renders them unable to give informed consent have been stated in the research protocol and the study has been approved by a research ethics committee. Consent to remain in the research must be obtained as soon as possible from the subject or a legally authorised representative.

31. The physician must fully inform the patient which aspects of their care are related to the research. The refusal of a patient to participate in a study or the patient's decision to withdraw from the study must never adversely affect the patient-physician relationship.

32. For medical research using identifiable human material or data, such as research on material or data contained in biobanks or similar repositories, physicians must seek informed consent for its collection, storage and/or reuse. There may be exceptional situations where consent would be impossible or impracticable to obtain for such research. In such situations the research may be done only after consideration and approval of a research ethics committee.

Use of Placebo

33. The benefits, risks, burdens and effectiveness of a new intervention must be tested against those of the best proven intervention(s), except in the following circumstances: Where no proven intervention exists, the use of placebo, or no intervention, is acceptable; or where for compelling and scientifically sound methodological reasons the use of any intervention less effective than the best proven one, the use of placebo, or no intervention is necessary to determine the efficacy or safety

of an intervention and the patients who receive any intervention less effective than the best proven one, placebo, or no intervention will not be subject to additional risks of serious or irreversible harm as a result of not receiving the best proven intervention. Extreme care must be taken to avoid abuse of this option.

Post-Trial Provisions

34. In advance of a clinical trial, sponsors, researchers and host country governments should make

provisions for post-trial access for all participants who still need an intervention identified as beneficial in the trial. This information must also be disclosed to participants during the informed consent process.

Research Registration and Publication and Dissemination of Results

35. Every research study involving human subjects must be registered in a publicly accessible database before recruitment of the first subject.

36. Researchers, authors, sponsors, editors and publishers all have ethical obligations with regard to the publication and dissemination of the results of research. Researchers have a duty to make publicly available the results of their research on human subjects and are accountable for the completeness and accuracy of their reports. All parties should adhere to accepted guidelines for ethical reporting. Negative and inconclusive as well as positive results must be published or otherwise made publicly available. Sources of funding, institutional affiliations and conflicts of interest must be declared in the publication. Reports of research not in accordance with the principles of this Declaration should not be accepted for publication.

Unproven Interventions in Clinical Practice

37. In the treatment of an individual patient, where proven interventions do not exist or other known

interventions have been ineffective, the physician, after seeking expert advice, with informed consent from the patient or a legally authorised representative, may use an unproven intervention if in the physician's judgement it offers hope of saving life, re-establishing health or alleviating suffering. This intervention should subsequently be made the object of research, designed to evaluate its safety and efficacy. In all cases, new information must be recorded and, where appropriate, made publicly available.

14. REFERENCES

[1] Weil RJ, Palmieri DC, Bronder JL, Stark AM, Steeg PS. Breast cancer metastasis to the central nervous system. Am J Pathol 2005;167:913-920.

[2] Lin NU and Winer EP. Brain metastases: the HER2 paradigm. Clin Cancer Res. 2007;13:1648-1655.

[3] Ufen MP, Köhne CH, Wischneswky M, et al. Metastatic breast cancer: are we treating the same patients as in the past? Ann Oncol 2014:25:95-100.

[4] Hammond ME, Hayes DF, Dowsett M, et al. American Society of Clinical Oncology/College Of American Pathologists guideline recommendations for immunohistochemical testing of estrogen and progesterone receptors in breast cancer. J Clin Oncol 2010;28:2784-2795

[5] Wolff AC, Hammond ME, Schwartz JN, et al. American Society of Clinical Oncology/College of American Pathologists guideline recommendations for human epidermal growth factor receptor 2 testing in breast cancer. J Clin Oncol 2007;25:118-145.

[6] Sanna G, Franceschelli L, Rotmensz N, et al. Brain metastases in patients with advanced breast cancer. Anticancer Res 2007;27:2865-2869.

[7] Berghoff A, Bago-Horvath Z, De Vries C, et al. Brain metastases free survival differs between breast cancer subtypes. Br J Cancer 2012;106:440-446.

[8] Soffietti R, Abacioglu U, Baumert B, et al. Diagnosis and treatment of brain metastases from solid tumors: guidelines from the European Association of Neuro-Oncology (EANO). Neuro Oncol 2017;19:162-174.

[9] Arvold ND, Lee EQ, Mehta MP, et al. Updates in the management of brain metastases. Neuro Oncol 2016;18:1043-1065,

[10] Berghoff AS, Schur S, Fureder LM, et al. Descriptive statistical analysis of a real life cohort of 2419 patients with brain metastases of solid cancers. ESMO Open 2016;1:e000024.

[11] Brastianos HC, Cahill DP, Brastianos PK. Systemic therapy of brain metastases. Curr Neurol Neurosci Rep 2015;15:518.

[12] Percy DB, Ribot EJ, Chen Y, et al. In vivo characterization of changing blood-tumor barrier permeability in a mouse model of breast cancer metastasis: a complementary magnetic resonance imaging approach. Invest Radiol 2011;46:718-25.

[13] Bachelot T, Romieu G, Campone M, et al. Lapatinib plus capecitabine in patients with previously untreated brain metastases from HER2-positive metastatic breast cancer (LANDSCAPE): a single-group phase 2 study. Lancet Oncol 2013;14:64-71.

[14] Freedman RA, Gelman RS, Wefel JS, et al. Translational Breast Cancer Research Consortium (TBCRC) 022: A Phase II Trial of Neratinib for Patients With Human Epidermal Growth Factor Receptor 2-Positive Breast Cancer and Brain Metastases. J Clin Oncol 2016;34:945-952.

[15] Freedman RA, Gelman RS, Anders CK, et al. TBCRC 022: A Phase II Trial of Neratinib and Capecitabine for Patients With Human Epidermal Growth Factor Receptor 2-Positive Breast Cancer and Brain Metastases. J Clin Oncol 2019;37:1081-1089.

[16] Murthy R, Borges VF, Conlin A, et al. Tucatinib with capecitabine and trastuzumab in advanced HER2-positive metastatic breast cancer with and without brain metastases: a non-randomised, open-label, phase 1b study. Lancet Oncol 2018;19:880-888.

[17] Hamilton E, Murthy R, Ferrario C, et al. Prolonged progression-free survival (PFS) in advanced HER2+ metastatic breast cancer with or without brain metastases: A pooled analysis of tucatinib phase 1b studies. Cancer Research 2018;78(suppl; abstr P5-20-01).

[18] Murthy RK, Loi S, Okines A, et al. Tucatinib, Trastuzumab, and Capecitabine for HER2-Positive Metastatic Breast Cancer. N Engl J Med 2020; 382:597-609.

[19] Kurihara H, Hamada A, Yoshida M, et al. (64)Cu-DOTA-trastuzumab PET imaging and HER2 specificity of brain metastases in HER2-positive breast cancer patients. EJNMMI Res 2015;5:8.

[20] Verma S, Miles D, Gianni L, et al. Trastuzumab emtansine for HER2-positive advanced breast cancer. N Engl J Med 2012;367:1783-1791.

[21] Krop IE, Lin NU, Blackwell K, et al. Trastuzumab emtansine (T-DM1) versus lapatinib plus capecitabine in patients with HER2-positive metastatic breast cancer and central nervous system metastases: a retrospective, exploratory analysis in EMILIA. Ann Oncol 2015;26:113-119.

[22] Bartsch R, Berghoff AS, Vogl U, et al. Activity of T-DM1 in Her2-positive breast cancer brain metastases. Clin Exp Metastasis 2015;32:729-737.

[23] Lin NU, Lee EQ, Aoyama H, et al. Response assessment criteria for brain metastases: proposal from the RANO group. Lancet Oncol 2015;16:e270-278.

[24] Jacot W, Pons E, Frenel JS, et al. Efficacy and safety of trastuzumab emtansine (T-DM1) in patients with HER2-positive breast cancer with brain metastases. Breast Cancer Res Treat 2016;157(2):307-318.

[25] Askoxylakis V, Ferraro GB, Kodack DP, et al. Preclinical Efficacy of Ado-trastuzumab Emtansine in the Brain Microenvironment. J Natl Cancer Inst 2015 7;108.

[26] Geraud A, Xu HP, Beuzeboc P, Kirova YM. Preliminary experience of the concurrent use of radiosurgery and T-DM1 for brain metastases in HER2-positive metastatic breast cancer. J Neurooncol 2017;131:69-72.

[27] Trastuzumab-deruxtecan Investigator's Brochure edition number 6.0; Dept. 9th 2019.

[28] Modi S, Saura C, Yamashita T, et al. Trastuzumab Deruxtecan in Previously Treated HER2-Positive Breast Cancer. N Engl J Med 2020; 382:610-621.

[29] von Minckwitz G, Huang CS, Mano MS, et al. Trastuzumab Emtansine for Residual Invasive HER2-Positive Breast Cancer. N Engl J Med 2019;380:617-628.

[30] Eisenhauer EA, Therasse P, Bogaerts J, et al. New response evaluation criteria in solid tumours: revised RECIST guideline (version 1.1). Eur J Cancer 2009;45:228-247.

[31] Simon R. Optimal two-stage designs for phase II clinical trials. Control Clin Trials 1989;10:1-10.

[32] Lin NU, Diéras V, Paul D, et al. Multicenter Phase II Study of Lapatinib in Patients With Brain Metastases From HER2-positive Breast Cancer. Clin Cancer Res 2009;15:1452-1459

[33] Oken MM, Creech RH, Tormey DC, et al. Toxicity and response criteria of the Eastern Cooperative Oncology Group. Am J Clin Oncol 1982;5:649-655.

[34] Available at: https://www.wma.net/policies-post/wma-declaration-of-helsinki-ethical-principles-for-medical-research-involving-human-subjects/; last accessed Sept. 10th 2019.
