# Supplementary material for: Trastuzumab deruxtecan in HER2-positive breast cancer with brain metastases: a single-arm, phase 2 trial
Source: Nat Med. 2022 Aug 8;28(9):1840–7. doi: 10.1038/s41591-022-01935-8 (PMC9499862; doi:10.1038/s41591-022-01935-8)
Supplement: Supplementary file 2 — Reporting Summary [file 41591_2022_1935_MOESM2_ESM.pdf]

nature portfolio

Corresponding author(s): Matthias Preusser

Last updated by author(s): Jun 29, 2022

Reporting Summary

Nature Portfolio wishes to improve the reproducibility of the work that we publish. This form provides structure for consistency and transparency in reporting. For further information on Nature Portfolio policies, see our Editorial Policies and the Editorial Policy Checklist.

Statistics

For all statistical analyses, confirm that the following items are present in the figure legend, table legend, main text, or Methods section.

n/a	Confirmed	
☐	☒	The exact sample size (*n*) for each experimental group/condition, given as a discrete number and unit of measurement
☐	☒	A statement on whether measurements were taken from distinct samples or whether the same sample was measured repeatedly
☐	☒	The statistical test(s) used AND whether they are one- or two-sided<br>*Only common tests should be described solely by name; describe more complex techniques in the Methods section.*
☐	☒	A description of all covariates tested
☐	☒	A description of any assumptions or corrections, such as tests of normality and adjustment for multiple comparisons
☐	☒	A full description of the statistical parameters including central tendency (e.g. means) or other basic estimates (e.g. regression coefficient) AND variation (e.g. standard deviation) or associated estimates of uncertainty (e.g. confidence intervals)
☐	☒	For null hypothesis testing, the test statistic (e.g. *F*, *t*, *r*) with confidence intervals, effect sizes, degrees of freedom and *P* value noted<br>*Give P values as exact values whenever suitable.*
☐	☒	For Bayesian analysis, information on the choice of priors and Markov chain Monte Carlo settings
☐	☒	For hierarchical and complex designs, identification of the appropriate level for tests and full reporting of outcomes
☒	☐	Estimates of effect sizes (e.g. Cohen's *d*, Pearson's *r*), indicating how they were calculated

Our web collection on statistics for biologists contains articles on many of the points above.

Software and code

Policy information about availability of computer code

Data collection	No software was used.
Data analysis	R 4.1.3. and IBM SPSS Statistic v28

For manuscripts utilizing custom algorithms or software that are central to the research but not yet described in published literature, software must be made available to editors and reviewers. We strongly encourage code deposition in a community repository (e.g. GitHub). See the Nature Portfolio guidelines for submitting code & software for further information.

Data

Policy information about availability of data

All manuscripts must include a data availability statement. This statement should provide the following information, where applicable:

- Accession codes, unique identifiers, or web links for publicly available datasets
- A description of any restrictions on data availability
- For clinical datasets or third party data, please ensure that the statement adheres to our policy

Pseudonymized participant data including baseline characteristics and results of primary, secondary, and exploratory endpoint analyses reported in this manuscript can be shared in compliance with current data protection regulations by the European Union. Data sharing requires a current and positive vote by the requestors competent ethics committee. All proposals should be directed to the corresponding author, and data requestors will need to sign a data access agreement with the Medical University of Vienna.

Human research participants

Policy information about [studies involving human research participants and Sex and Gender in Research.](#)

Reporting on sex and gender	Female (N=14) and male (N=1) adult patients were included in this study.
Population characteristics	Female and male adult patients with histologically confirmed HER2-positive breast cancer (defined by a score of 3+ on immunohistochemistry and/or HER2/neu gene amplification) and newly diagnosed locally untreated BM or BM progressing after prior local therapy, prior exposure to trastuzumab and pertuzumab, and no indication for immediate local therapy could be included. Patients were required to have measurable disease as defined by Response Assessment in Neuro-Oncology (RANO)-BM criteria [ ],Karnofsky Performance Status >70%, Eastern Cooperative Oncology Group Performance Status <2, indication for systemic anti-HER2 treatment, and an estimated life expectancy of at least three months. Adequate cardiac, bonemarrow, liver and kidney function were required. Prior T-DM1 treatment was allowed but was not mandated. A total number of fifteen planned patients (14 female, one male) had received at least once cycle of trastuzumab deruxtecan; 60% had brain metastases progressing after prior local therapy, 60% had received prior T-DM1. The median time from the last local intervention to study inclusion in patients with prior local therapy was 13 months (range 5-65 months). Median age upon inclusion was 69 years (30-76 years), Eastern Cooperative Oncology Group (ECOG) performance status was 0 in 60% of patients and 40% had neurological symptoms at baseline. Twelve patients had hormone-receptor positive and HER2-positive disease and brain-only disease was present in two participants.
Recruitment	Patients were recruited accroding to predefined inclusion and exclusion criteria from consecutive patients presenting to the study site. The indication for sytemic therapy was reviewed by multidisiplinary experts in a regular tumor board and according to international treatment guidelines. Despite the strong biological rational, the stringent response evaluation by RANO-BM criteria with central response assessment, the availability of biomarkers, and extensive quality-of-life evaluation, the study is limited by the unrandomized phase II design and the small sample size and thus, an inclusion bias cannot be fully excluded. TUXEDO-1 enrolled not only HER2-positive/ER-negative tumours, but also HER2-positive/ER-positive cases, which show later recurrences and a less aggressive course of disease. However, response to trastuzumab deruxtecan therapies do not differ between these subtypes in the DESTINY-Breast01 trial and thus the overrepresentation of patients with luminal disease is not a likely reason for the high response rate observed in the study population.
Ethics oversight	Local ethics committee of the Medical University of Vienna (EC number 1359/2020).

Note that full information on the approval of the study protocol must also be provided in the manuscript.

Field-specific reporting

Please select the one below that is the best fit for your research. If you are not sure, read the appropriate sections before making your selection.

☒ Life sciences ☐ Behavioural & social sciences ☐ Ecological, evolutionary & environmental sciences

For a reference copy of the document with all sections, see [nature.com/documents/nr-reporting-summary-flat.pdf](#)

Life sciences study design

All studies must disclose on these points even when the disclosure is negative.

Sample size	A Simon's two-stage design was applied. An intracranial RR exceeding 60% was considered as indicating clinically relevant activity in this patient population. The null hypothesis that the true RR was 26% was tested against a one-sided alternative. Based upon these assumptions, six patients were to be accrued in the first stage. If at least three responses were observed in the first stage, nine additional patients were to be accrued for a total number of 15 patients.
Data exclusions	Main exclusion criteria included presence of malignancies other than BC, presence of leptomeningeal disease by local assessment at baseline, and severe pre-existing pulmonary disease. The full list of inclusion- and exclusion criteria is available in the manuscript text and study protocol.
Replication	The project is a prospective clinical trial and thus no replication was foreseen within the scope of this study. Replication of the results requires further clinical trials.
Randomization	This is a prospective, open-label, single-arm phase 2 trial.
Blinding	This is a prospective, open-label, single-arm phase 2 trial.

Reporting for specific materials, systems and methods

We require information from authors about some types of materials, experimental systems and methods used in many studies. Here, indicate whether each material, system or method listed is relevant to your study. If you are not sure if a list item applies to your research, read the appropriate section before selecting a response.

Materials & experimental systems

n/a	Involved in the study
☒	☐ Antibodies
☒	☐ Eukaryotic cell lines
☒	☐ Palaeontology and archaeology
☒	☐ Animals and other organisms
☐	☒ Clinical data
☒	☐ Dual use research of concern

Methods

n/a	Involved in the study
☒	☐ ChIP-seq
☒	☐ Flow cytometry
☒	☐ MRI-based neuroimaging

Clinical data

Policy information about clinical studies

All manuscripts should comply with the ICMJE guidelines for publication of clinical research and a completed CONSORT checklist must be included with all submissions.

Clinical trial registration	NCT04752059
Study protocol	The full trial protocol is included in the submission.
Data collection	Recruitment and data collection was performed between July 2020 until July 2021 at the Divison of Oncology, Medical University of Vienna, Austria.
Outcomes	Primary endpoint was the rate of best responses of BM at any radiological assessment after the administration of at least one cycle of T-DXd according to the RANO-BM criteria as determined by central assessment. Secondary endpoints consisted of Clinical Benefit Rate CNS (defined by RANO-BM), extracranial RR (according to RECIST 1.1 criteria), PFS, time-to-WBRT, OS, safety and QoL.
